# MorphoSONIC: A morphologically structured intramembrane cavitation model reveals fiber-specific neuromodulation by ultrasound

**DOI:** 10.1016/j.isci.2021.103085

**Published:** 2021-09-06

**Authors:** Théo Lemaire, Elena Vicari, Esra Neufeld, Niels Kuster, Silvestro Micera

**Affiliations:** 1 Bertarelli Foundation Chair in Translational Neuroengineering, Center for Neuroprosthetics and Institute of Bioengineering, School of Engineering, École Polytechnique Fédérale de Lausanne (EPFL), 1202 Lausanne, Switzerland; 2 Biorobotics Institute, Scuola Superiore Sant’Anna (SSSA), 56127 Pisa, Italy; 3 Foundation for Research on Information Technologies in Society (IT’IS), 8004 Zurich, Switzerland; 4 Department of Information Technology and Electrical Engineering, Swiss Federal Institute of Technology (ETH) Zurich, 8092 Zurich, Switzerland

**Keywords:** Ultrasound technology, Neuroscience, Computer modeling

## Abstract

Low-Intensity Focused Ultrasound Stimulation (LIFUS) holds promise for the remote modulation of neural activity, but an incomplete mechanistic characterization hinders its clinical maturation. Here we developed a computational framework to model intramembrane cavitation (a candidate mechanism) in multi-compartment, morphologically structured neuron models, and used it to investigate ultrasound neuromodulation of peripheral nerves. We predict that by engaging membrane mechanoelectrical coupling, LIFUS exploits fiber-specific differences in membrane conductance and capacitance to selectively recruit myelinated and/or unmyelinated axons in distinct parametric subspaces, allowing to modulate their activity concurrently and independently over physiologically relevant spiking frequency ranges. These theoretical results consistently explain recent empirical findings and suggest that LIFUS can simultaneously, yet selectively, engage different neural pathways, opening up opportunities for peripheral neuromodulation currently not addressable by electrical stimulation. More generally, our framework is readily applicable to other neural targets to establish application-specific LIFUS protocols.

## Highlights

• Ultrasound Stimulation (US) of peripheral circuits lacks biophysical understanding
• We developed a modeling framework to study US in morphologically-realistic neurons
• Simulations reveal that US can selectively recruit myelinated and/or unmyelinated fibers
• Multiplexed US could modulate distinct fiber populations simultaneously and independently

## Graphical abstract

**Figure:**
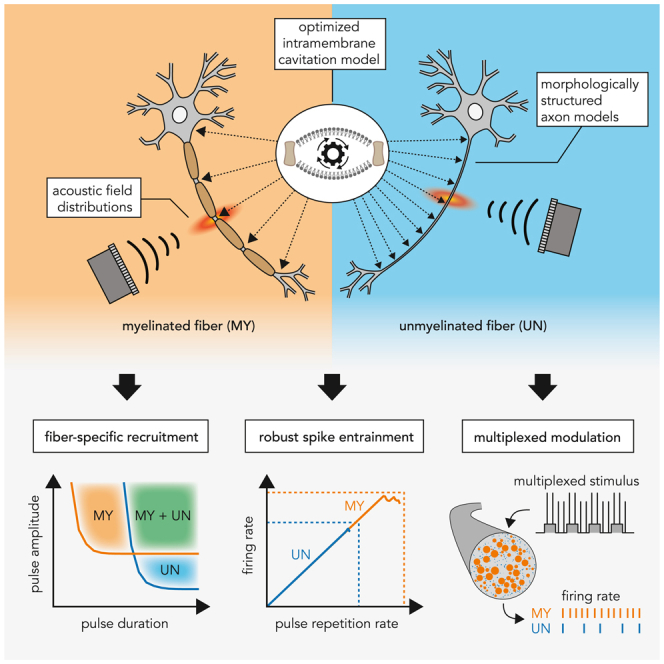

## Introduction

Ultrasound-based approaches have been increasingly adopted over the past decades for a variety of noninvasive therapeutic interventions (Escoffre and Bouakaz, 2016). These therapies rely on the mechanical nature of acoustic waves that propagate efficiently through biological tissue and can be accurately steered to concentrate mechanical energy within small volumes (∼mm^3^) around deep anatomical targets. In recent years, several in vitro and in vivo studies have shown that such acoustic waves can also be used to reversibly modulate the activity of various neural targets with remarkable spatial accuracy (Blackmore et al., 2019). These findings have propelled the development of Low-Intensity Focused Ultrasound Stimulation (LIFUS) as a novel technology to achieve noninvasive, selective, and reversible neuromodulation of virtually any neural structure.

Yet, despite a decade of intense investigation, several open issues have impeded the development of LIFUS as a clinically relevant technology. The variety of known physical effects of acoustic waves in biological tissue implies a wide range of possibilities for how neurons may translate mechanical energy into electrical responses, including membrane conformational changes because of thermodynamic coupling (Heimburg and Jackson, 2005), flexoelectricity, (Petrov, 2002) and mechanosensitive channels activation (Tyler, 2011). At the same time, distinguishing these candidate mechanisms in experimental settings and establishing their predominance over the multidimensional LIFUS parameter space remains a challenge. Consequently, it is difficult to provide a mechanistic perspective that would clarify and guide the heterogeneous and sometimes conflicting collection of neuromodulatory effects (excitatory and inhibitory, short and long term, localized and large-scale, reversible and permanent) obtained across animal models, neural targets, and experimental designs.

In light of these challenges, computational approaches have become helpful tools to increase the understanding of ultrasound-neuron interactions, as they allow a specific candidate mechanism to be examined. A significant effort made by (Plaksin et al., 2014), who introduced the ***N****euronal* ***I****ntramembrane* ***C****avitation* ***E****xcitation* (NICE) model, described a candidate mechanism in which LIFUS induces the cavitation of specific phospholipidic structures (so-called bilayer sonophores), thereby dynamically altering membrane capacitance and triggering action potentials. This model predicts cell-type-specific LIFUS responses of cortical and thalamic neurons (Plaksin et al., 2016) that correlate indirectly with a range of empirical results obtained in the central nervous system (CNS) (Kim et al., 2012; King et al., 2013; Tufail et al., 2011; Yoo et al., 2011).

The NICE model, however, entails a significant numerical stiffness that has so far limited its applications to point-neuron studies (Plaksin et al., 2014, 2016; Tarnaud et al., 2019) that could not address physiologically relevant questions, such as the influence of intracellular axial coupling and morphological inhomogeneity on neuronal responses, the spatiotemporal dynamics of those responses, and the impact of spatial features of the acoustic field on excitability (as is the case for electrical stimulation). Hence, a multi-compartment model of intramembrane cavitation incorporating morphological details would be highly beneficial to increase our understanding of LIFUS neuromodulation by intramembrane cavitation in a more realistic setting.

In a recent study, we developed a *multi-****S****cale* ***O****ptimized* ***N****euronal* ***I****ntramembrane* ***C****avitation* (SONIC) model that alleviates the numerical stiffness of the NICE model by integrating the coarse-grained evolution of effective electrical variables as a function of a precomputed, cycle-averaged impact of the oscillatory mechanical system (Lemaire et al., 2019), thereby drastically reducing computational costs while maintaining numerical accuracy. Building on this effective paradigm, we present *morphoSONIC*, a novel framework to simulate intramembrane cavitation into morphologically structured neuron models. This framework leverages the optimized modeling and numerical integration pipelines of the NEURON simulation environment (Hines and Carnevale, 1997), and provides an alternative implementation of its internal cable representation as a hybrid (charge and voltage casted) electrical circuit that is numerically compatible with the SONIC model.

Specifically, we exploit this framework to investigate the mechanisms of ultrasonic neuromodulation in myelinated and unmyelinated peripheral fibers, using previously validated single-cable axon models (Reilly et al., 1985; Sundt et al., 2015). First, we provide an in-depth analysis of predicted LIFUS neuronal responses and recruitment mechanisms in both fiber types. Second, we characterize their LIFUS excitability by evaluating strength-duration (SD) “signatures” across a wide range of model and stimulation parameters and compare those signatures to those traditionally obtained with electrical stimulation. Third, we identify key morphological features underlying the distinct LIFUS sensitivities of myelinated and unmyelinated axons. Fourth, we analyze cell-type-specific neuronal responses upon repeated acoustic exposure and identify pulsing regimes yielding a robust modulation of fiber spiking activity. Finally, we propose a new type of pulsing protocol to achieve the concurrent, yet independent, modulation of myelinated and unmyelinated axons within heterogeneous nerve bundles.

## Results

**Figure 1:**
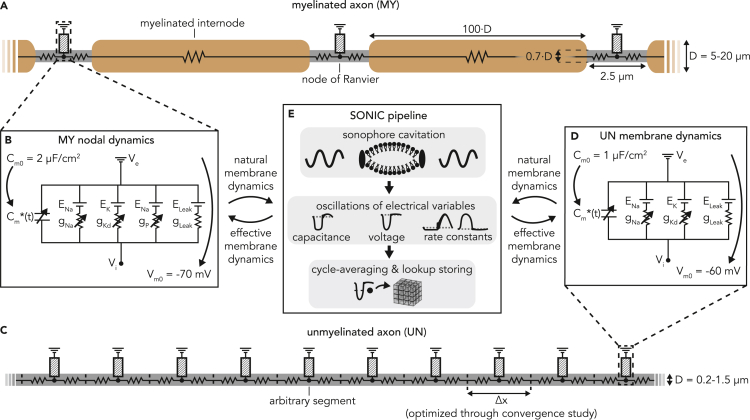
Morphology, biophysics and incorporation of the SONIC paradigm in myelinated and unmyelinated axon models (A) Schematic of the myelinated axon model morphology. (B) Electrical circuit representation of the membrane dynamics at the nodes of Ranvier. (C and D) Equivalent morphological and biophysical descriptions of the uniform unmyelinated axon. (E) Schematic diagram showing the incorporation of the SONIC paradigm into the axon models.

**Figure 2:**
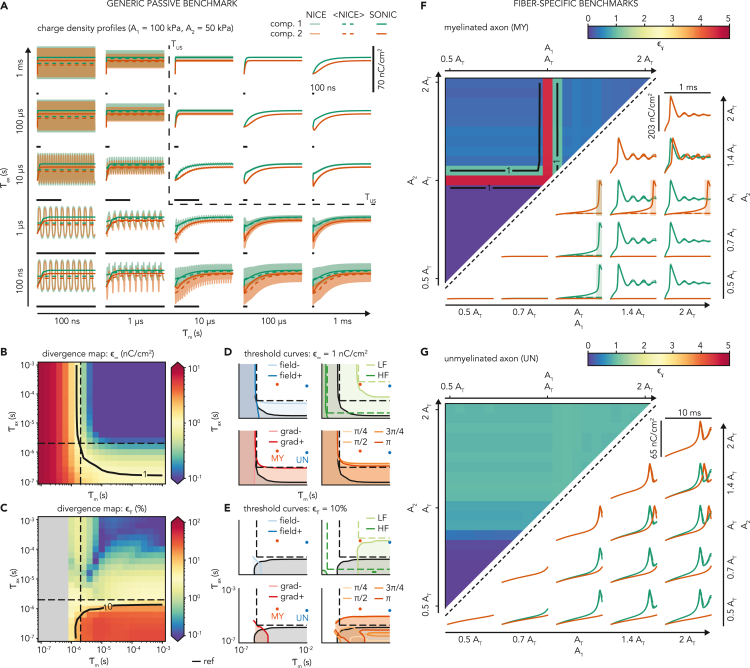
Evaluation of the SONIC paradigm accuracy in two-compartment benchmark models (A) Temporal evolution of charge density from temporally detailed (solid shaded lines) and cycle-averaged (dashed lines) NICE solutions as well as SONIC (solid lines) solutions for a reference ultrasound stimulus (*f*_*US*_ = 500 kHz, A_1_ = 100 kPa, A_2_ = 50 kPa), shown for various combinations of membrane and axial time constants in a two-compartment passive model (C_m0_ = 1 μF/cm2, E_Leak_ = −70 mV). For each combination, the horizontal scale bar indicates 100 ns. Black dashed lines indicate the acoustic stimulus period (*T*_*US*_). (B) Color map showing the absolute steady-state deviation of charge density $\varepsilon_{\infty }$ between SONIC and cycle-averaged NICE solutions in the passive model as a function of the model’s electrical time constants, for the same ultrasound stimulus. The maximal deviation across model compartments is reported for each combination. The acoustic period (dashed lines) is indicated, as well as the threshold curve for a critical deviation level of 1 nC/cm^2^ (solid line). (C) Color map showing the average absolute deviation $\varepsilon_{\tau }$ between SONIC and cycle-averaged NICE charge density curves normalized to a unit interval, as a function of the model’s time constants, for the same ultrasound stimulus. The maximal deviation across compartments is reported, and a threshold curve (solid line) indicates a critical deviation level of 10%. (D) Steady-state SONIC threshold curves $\left(\varepsilon_{\infty }=1\;\text{nC}/{\text{cm}}^{2}\right)$ and divergent areas $\left(\varepsilon_{\infty }>1\;\text{nC}/{\text{cm}}^{2}\right)$ across the time constant space for the independent variations of stimulus amplitude (top left), frequency (top right), amplitude gradient (bottom left) and phase gradient (bottom right). The reference condition is indicated in black. Dashed lines indicate the stimulus period for each carrier frequency. Solid points indicate the location of fiber-specific passive properties in this 2-dimensional space. (E) Transient SONIC threshold curves ($\varepsilon_{\tau }=10\%$) and deviation areas ($\varepsilon_{\tau }>10\%$) across the time constant space, for identical variations of stimulus parameters as in (D). (F) Charge density profiles and associated color map showing the 2-dimensional gamma distance between profiles from SONIC and cycle-averaged NICE solutions in a two-compartment benchmark model of the myelinated axon for various combinations of acoustic pressure phasor amplitudes (*f*_*US*_ = 500 kHz, 1 ms stimulus). Color codes on the map report the maximal gamma distance across compartments for each combination, with threshold curves (black lines) indicating a critical deviation level ($\varepsilon_{\gamma }=1$). Shaded areas on the plots indicate time intervals of gamma deviation ($\varepsilon_{\gamma }>1$). (G) Equivalent charge density profiles and gamma distance color map in a two-compartment benchmark model of the unmyelinated axon (10 ms stimulus).

We investigated the mechanisms of US neuromodulation by intramembrane cavitation in peripheral nerve fibers, as these structures represent privileged, accessible neuromodulation targets. Moreover, multiple studies have demonstrated the paramount influence of their morphology on the resulting excitability by electric fields (McNeal, 1976; Rattay, 1986). As such, they are natural candidates for the study of LIFUS effects in morphologically structured models. To this aim, we used single-cable axonal representations of myelinated and unmyelinated fibers (Reilly et al., 1985; Sundt et al., 2015) (SENN and Sundt model, respectively, Figure 1) allowing for a numerically valid incorporation of the SONIC paradigm (see Figure 2) while maintaining a certain level of morphological realism.

Given the multiplicity of parameters in the SONIC model and the remaining uncertainty regarding key variables (mainly sonophore dimensions and density) and their distribution across morphological structures, we opted here to constrain this input space in order to restrict our analysis to an acceptable dimensionality. Thus, in all simulations, we considered that sonophores are absent from myelinated model sections, and assumed a uniform sonophore density across the remaining compartments (set by default to f_s_ = 80% - a geometrically plausible value yielding robust excitability in cortical point-neuron models; see (Lemaire et al., 2019, Figure 10). We also chose a typical sonophore radius (a = 32 nm) already used in previous studies (Lemaire et al., 2019; Plaksin et al., 2014, 2016), unless stated otherwise. Internal SONIC model parameters were also defined as in (Lemaire et al., 2019). Importantly, these parameters were adopted as is, without returning or post-hoc adjustments.

Similarly, the expansion of acoustic simulations to spatially-extended structures also expands the dimensionality of the stimulus parameter space: beyond the carrier frequency (*f*_*US*_) and pulsing protocol, amplitudes (A) and phases (*ϕ*) of the pressure phasor affecting each model compartment must be chosen, suggesting the need to compute acoustic pressure distributions in the model region. Here again, a wide number of distributions could be considered. Thus, to constrain the stimulus input space, we considered the simple (yet realistic and relevant) case of a single-element planar transducer pointing normally toward a peripheral fiber bundle lying inside a water-like acoustic medium (soft tissue or liquid) at the transducer focal distance, and used a detailed physics model of acoustic propagation to compute the resulting nature of acoustic distributions along fibers (Figure S1A). Simulations revealed that under this particular orthogonal arrangement, spatial profiles of peak acoustic pressure along the fiber are well approximated by Gaussian distributions (the width of which depends on source parameters such as transducer radius and carrier frequency), whereas phase gradients remain small in the focal region (Figures S1A–S1C). Hence, in the following sections, we assumed Gaussian-shaped pressure exposure along fibers. Finally, in order to further constrain the input space, we considered in most of our analyses a single ultrasound carrier frequency (*f*_*US*_ = 500 kHz), lying comfortably within the theoretical “likelihood” range for intramembrane cavitation (Krasovitski et al., 2011).

### The SONIC paradigm is numerically accurate in multi-compartment axon models

Two recent studies have shown that in multi-compartment structures, the presence of large axial conductances could introduce significant intra-cycle charge redistribution mechanisms during intramembrane cavitation, thereby inducing a significant numerical deviation of the SONIC paradigm from its temporally detailed NICE counterpart (Tarnaud et al., 2020, 2021). Thus, to establish the conditions of this deviation and whether it applies to the models of this study, we performed a series of numerical simulations in two-compartment benchmark models, allowing the simulation of temporally detailed intramembrane cavitation dynamics in each compartment with reasonable computational effort. Using these models, we simulated ultrasound-evoked responses under both the detailed NICE and the coarse-grained SONIC paradigms, and evaluated SONIC accuracy by comparing charge density profiles from SONIC solutions to those of cycle-averaged NICE solutions (considered as “numerical ground truth”).

First, we investigated the influence of passive model properties on the accuracy of the SONIC paradigm in predicting sub-threshold depolarization, a critical aspect of neuronal responses. To this end, we used a two-compartment model depleted of nonlinear membrane currents (see STAR Methods), which responds to acoustic perturbation with a rapid charge build-up converging exponentially toward a steady-state plateau. This stereotypical dynamics allowed to evaluate SONIC accuracy in simulating both transient and long-lasting neural dynamics, by measuring (i) the average relative transient difference between unit-interval-normalized charge density profiles ($\varepsilon_{\tau }$, in %) and (ii) the absolute steady-state difference in charge density ($\varepsilon_{\infty }$, in nC/cm^2^), respectively (see STAR Methods). We thus evaluated these two difference metrics while independently varying the model membrane and axial time constants ($\tau_{m}$ and $\tau_{ax}$, respectively). These two parameters are directly derived from the biophysical model properties and provide quantitative estimates of the time taken by leakage and axial currents to respond to variations in transmembrane and longitudinal intracellular voltage gradients, respectively.

For a reference condition of spatially-varying acoustic field (carrier frequency *f*_*US*_ = 500 kHz, pressure phasor of uniform phase and amplitudes *A*_*1*_ = 100 kPa and *A*_*2*_ = 50 kPa in compartments 1 and 2 respectively), SONIC deviations from the NICE reference showed a clear dependency on both axial and membrane time constants (Figures 2A–2C). Weak electrical conductances (i.e., long time constants) yield a slowly responding electrical system that filters out the intra-cycle capacitance and voltage oscillations and evolves smoothly as a function of the cycle-averaged value of these variables. Under these conditions, the SONIC paradigm performs very well and accurately captures both the transient and steady-state phases of the neural response, resulting in a large subspace of SONIC reliability ($\varepsilon_{\infty }<1\;\text{nC}/{\text{cm}}^{2}$ and $\varepsilon_{\tau }<10\%$). Conversely, strong electrical conductances (i.e., short time constants) increase the sensitivity of the electrical system to the oscillatory mechanical drive to a point that currents instantaneously “convert” part of the capacitive displacement energy into fast charge redistribution during an acoustic period, thereby impacting the net charge variation over that period. We can differentiate two distinct mechanisms of intra-cycle charge redistribution. On the one hand, leakage currents opposing intra-cycle deviations from reversal potentials create a transmembrane charge redistribution that reduces the net charge increase at each cycle, hindering the slow scale charge build-up in each compartment and affecting both transient and steady-state regimes of the resulting membrane dynamics. On the other hand, intracellular currents opposing intra-cycle voltage gradients create an axial charge redistribution across the two compartments that reduces the net charge gradient achieved over a cycle, ultimately limiting the magnitude of effective charge density gradients over time. Interestingly, this axial redistribution mechanism affects membrane dynamics more profoundly during the transient regime of charge build up, and has a rather limited impact on the steady-state regime for the chosen stimulus parameters. As neither of these redistribution mechanisms are captured by the SONIC paradigm, fast membrane and axial time constants both produced subspaces of SONIC deviation ($\varepsilon_{\infty }>1\;\text{nC}/{\text{cm}}^{2}$ and $\varepsilon_{\tau }>10\%$). Interestingly, significant SONIC deviations emerged as the membrane and/or axial time constant approached the order of magnitude of the acoustic period.

To generalize our observations, we replicated this deviation analysis while varying independently intrinsic features of the imposed acoustic stimulus, and analyzed the impact of these variations on the resulting SONIC deviation bifurcations (Figures 2D and 2E). First, we modulated pressure phasor amplitudes in both compartments by a constant factor: a uniform two-fold reduction (A_1_ = 50 kPa, A_2_ = 25 kPa, *field-*condition) shifted the steady-state bifurcation toward slower time constants and kept transient deviations below 10% across the entire domain, whereas a uniform two-fold increase (A_1_ = 200 kPa, A_2_ = 100 kPa, *field +* condition) almost entirely eliminated the impact of the model axial properties on SONIC accuracy. These somewhat counter-intuitive results can be explained by the nonlinear dependence of the sonophore cavitation amplitude on the imposed acoustic pressure phasor amplitude, which results in stronger variations in the 25–50 kPa range than in the 100–200 kPa range, thereby amplifying intra-cycle axial charge redistribution and causing SONIC inaccuracy at lower field amplitudes. Next, we modulated the carrier frequency of the stimulus: higher frequencies (*f*_*US*_ = 4 MHz, *HF* condition) and lower frequencies (*f*_*US*_ = 20 kHz, *LF* condition) shifted the transient and steady-state bifurcations toward faster or slower time constants, respectively, across both the membrane and axial dimensions. Subsequently, we varied the pressure phasor amplitude gradient across the model compartments: an increased pressure gradient (A_1_ = 200 kPa, A_2_ = 25 kPa, *gradient +* condition) amplified intra-cycle axial charge redistribution, thereby shifting the steady-state bifurcation toward slower axial time constants. Conversely, a null pressure gradient (A_1_ = A_2_ = 75 kPa, *gradient −* condition) completely decoupled the SONIC accuracy from axial time constants. Finally, while keeping a null pressure phasor amplitude gradient, we analyzed the impact of various acoustic phase shifts between the compartments. Unsurprisingly, increasing phase shifts resulted in a progressive expansion of both the transient and steady-state SONIC inaccuracy subspaces toward longer model time constants, with a maximal deviation observed for a phase shift Δφ=3π/4. Notably, across all amplitudes, frequencies and gradients of the acoustic pressure phasor, SONIC deviations from the NICE reference are restricted to specific sub-regions where at least one of the two time constants is shorter than the stimulus periodicity, yielding a robust domain of SONIC accuracy that can be mathematically expressed as $\left\{\tau_{m},\;\tau_{ax}\right\}>1/f_{US}$ (only major phase differences can result in deviations slightly above 1/*f*_*US*_ in the axial dimension). In other words, these findings suggested that the critical condition for the SONIC approximation to reliably reproduce the full NICE model is that the membrane and axial time constants (as obtained from the model biophysical properties) should be longer than the acoustic period. Importantly, the passive properties of both axon models used in this study satisfied this criterion, except at very low drive frequencies (*f*_*US*_ < 50 kHz), which are significantly lower than those employed in typical neuromodulation studies (Downs et al., 2018; King et al., 2013; Kubanek et al., 2020; Lee et al., 2020; Menz et al., 2019; Tyler et al., 2008; Wright et al., 2017).

Second, we investigated the applicability of the SONIC paradigm for the particular axon models used in this study, using two-compartment models with axon-specific morphological properties and full membrane ionic populations. The presence of highly nonlinear spiking dynamics in these models precludes the use of direct comparison metrics (such as the root-mean-square error), which are overly sensitive to small temporal offsets between two curves. Therefore, to accommodate for this intricate dynamics, SONIC deviation $\varepsilon_{\gamma }$ was quantified using the γ-index (Low et al., 1998), i.e., the point-wise minimal multidimensional time and charge density Euclidean distance between the cycle-averaged NICE and SONIC solutions, using appropriate temporal and charge density tolerances based on model-specific spiking features (see STAR Methods). We thus evaluated SONIC deviation across a symmetric two-dimensional space (for the two compartments) of pressure phasor amplitudes ranging from half to double each model’s excitation threshold (determined with the SONIC method), with a uniform carrier frequency (*f*_*US*_ = 500 kHz).

As expected, subthreshold pressure phasor amplitude distributions triggered passive build-ups in charge density in both models that were accurately captured by the SONIC paradigm (Figures 2F and 2G). Narrow bands of SONIC inaccuracy ($\varepsilon_{\gamma }>1$) appear in the myelinated case when either compartment is just at the excitation threshold, because of the sensitivity of spike initiation to small differences in initial build-up between the two paradigms; however, those differences vanish already at a small distance from the threshold. In the unmyelinated case, the slower intrinsic membrane dynamics enabled a robust SONIC accuracy across the entire explored pressure phasor amplitude range ($\varepsilon_{\gamma }<1$).

Taken together, these findings revealed that in the context of single-cable peripheral axon models, the SONIC paradigm offers acceptable numerical accuracy with respect to the temporally-detailed NICE paradigm across the entire LIFUS parameter space. As such, it can be reliably applied to investigate intramembrane cavitation in those models.

### LIFUS modulates membrane capacitance to excite peripheral nerve fibers

After verifying the applicability of the SONIC paradigm in multi-compartment axon models (Figure 2), we simulated the “typical” predicted responses of myelinated and unmyelinated axons to ultrasound stimulation. To this end, we selected standard axon models using representative diameters for each axon population (10 μm and 0.8 μm for myelinated and unmyelinated fibers, respectively), as well as a standard acoustic exposure (5 mm-wide distribution of acoustic pressure phasor amplitudes with a spatial peak of 120 kPa) qualitatively equivalent to that generated by a 10 mm diameter planar transducer at 500 kHz (see Figure S1B). We also used fiber-dependent pulse durations (1 ms and 10 ms for the myelinated and unmyelinated fibers, respectively), which are within their respective rheobase regime (see next section). To better identify mechanisms of axonal recruitment by LIFUS pulses, we quantified the time required to reach a build-up of normalized charge density $\left(Q_{m,norm}\left(t\right)=Q_{m}\left(t\right)/C_{m0}\right)$ of 5 mV in the central compartment for each response and computed the contribution of each individual current to this initial build-up.

**Figure 3:**
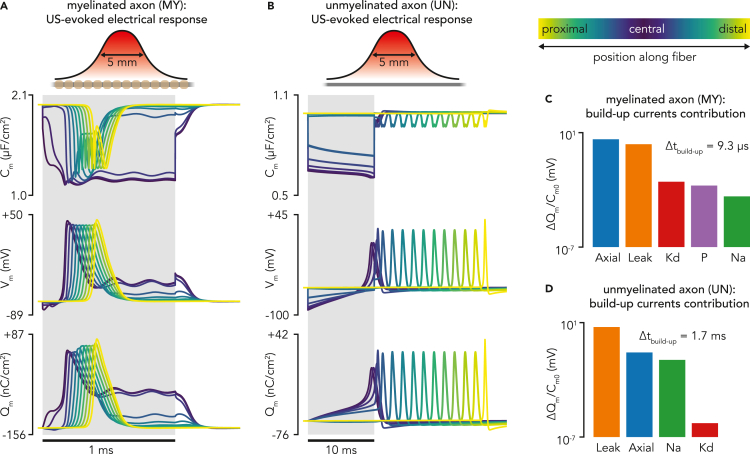
Typical responses of myelinated and unmyelinated axon models to a single LIFUS pulse (A) Time profiles of effective membrane capacitance, effective membrane potential, and effective membrane charge density across compartments during a typical response of a myelinated axon to a 1 ms sonication (500 kHz frequency, 5 mm-wide Gaussian pressure distribution aligned on the fiber with a spatial peak of 120 kPa). (B) Equivalent time profiles during the typical response of an unmyelinated axon to a 10 ms sonication (identical pressure distribution as in (A)). (C) Quantification of the membrane and axial current’s contribution to the first 5 mV of normalized charge build-up in the fiber central compartment. (D) Equivalent quantification for the response of the unmyelinated axon’s central compartment.

For both models, the sonication pulse onset generated instantaneous drops in effective membrane capacitance in the axon compartments, whose magnitude increased with the amplitude of the local acoustic pressure, thereby amplifying the absolute value of transmembrane voltage and inducing hyperpolarization (Figures 3A and 3B). Because of the Gaussian distribution of acoustic pressure along the axon, central compartments experienced a stronger hyperpolarization than peripheral ones, which introduced a longitudinal gradient in transmembrane voltage along the fiber.

At the central compartment where hyperpolarization is the largest, leakage currents arose to bring transmembrane voltage toward the leakage reversal potential, thereby inducing a build-up in local charge density. The considerably higher density of leakage channels in the Ranvier node compared to the unmyelinated membrane $\left(g_{leak}^{SENN}\approx 300\;g_{leak}^{Sundt}\right)$ induced much larger leakage currents (Figures 3C and 3D). Moreover, significant axial currents also arose in the myelinated axon, driven by large voltage gradients between the central and neighboring Ranvier nodes. Together, these two depolarizing currents yielded a much faster membrane charge build-up in the myelinated axon, yielding shorter response latencies. These differences are reflected in the times required to achieve a normalized charge build-up of 5 mV in the central compartment (9 μs and 1.7 ms for myelinated and unmyelinated fibers, respectively).

For a long enough sonication, membrane charge density increased until a spiking threshold was reached, prompting the opening of Sodium ion channels and thereby triggering an AP in the central compartment that started traveling bidirectionally toward the axon extremities. As expected, both axons exhibited marked differences in conduction velocities: fast saltatory conduction in the large diameter myelinated axon allowed the AP to reach the extremities of the axon in less than 1 ms, whereas that process took more than 10 ms in the slowly conducting unmyelinated axon. As the sonication outlasted the AP duration in the myelinated axon, affected nodes transitioned into a “plateau potential” regime (stabilization of membrane charge density around a depolarized value).

Finally, the sonication offset removed the mechanical membrane perturbation, and effective membrane capacitances instantaneously reverted to their resting values, triggering a rapid reduction in transmembrane voltage magnitudes. The myelinated axon then simply repolarized back to its equipotential resting state, whereas the AP propagated toward peripheral extremities in the unmyelinated axon.

The effect of electromechanical coupling was visible across neuronal responses. During the sub-threshold charge build-up, the decrease in electric pressure (a constraining force on the bilayer sonophore, proportional to $Q_{m}^{2}$) amplified membrane deflections, which further reduced the effective membrane capacitance (an effect more pronounced on the myelinated axon). In addition, the propagating spike induced a wave of time-varying electric pressure that also modulated the effective membrane capacitance.

### LIFUS can selectively recruit myelinated and unmyelinated fibers

The analysis of “typical” LIFUS-evoked responses revealed that ultrasonic axon recruitment requires the membrane charge density to be brought locally above a spiking threshold to engage voltage-gated channels. Yet, the underlying mechanisms eliciting this charge build-up differ significantly from those of electrical stimulation (McNeal, 1976; Rattay, 1986). Therefore, we aimed to determine if the two stimulation modalities could produce distinct excitability patterns. To this end, we computed excitation thresholds for various pulse durations ranging from 10 μs to 1 s (using binary search procedures) to construct strength-duration (SD) curves.

**Figure 4:**
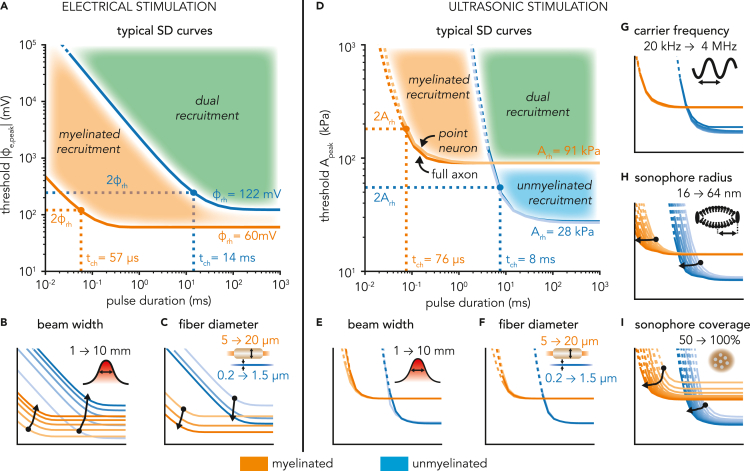
Comparison of strength-duration curves of myelinated and unmyelinated axons upon electrical and ultrasonic stimulation (A) SD curves of representative myelinated (10 μm diameter, in orange) and unmyelinated (0.8 μm diameter, in blue) axons, depicting the threshold absolute peak extracellular voltage required to elicit fiber excitation as a function of pulse duration, for a characteristic 5 mm wide Gaussian extracellular voltage distribution. Rheobase and chronaxie values of each curve are indicated, as well as distinct areas of fiber recruitment. (B) SD curves of representative myelinated and unmyelinated axons for Gaussian extracellular voltage distributions of varying widths (1–10 mm). Arrows indicate the translation of the chronaxie point in the SD space for increasing stimulus width. (C) SD curves both fiber types upon stimulation with a characteristic voltage distribution, for varying fiber diameters within the physiological range of each population (myelinated: 5 to 20 μm, unmyelinated: 0.2 to 1.5 μm). Arrows indicate the translation of the chronaxie point in the SD space for increasing fiber diameter. (D) SD curves of representative myelinated and unmyelinated axons, depicting the threshold peak acoustic pressure amplitude required to elicit fiber excitation as a function of pulse duration, for a characteristic 5 mm wide Gaussian acoustic pressure distribution and ultrasound frequency (f_US_ = 500 kHz), using typical values of sonophore radius (a = 32 nm) and sonophore coverage fraction (f_s_ = 80%) in the model’s compartments. SD curves using equivalent “node” models located under the stimulus peak are also indicated (light blue and orange curves), as well as rheobase and chronaxie values of each curve, and distinct areas of fiber recruitment. (E) SD curves of representative myelinated and unmyelinated axons with typical ultrasound frequency and sonophore parameters for Gaussian pressure distributions of varying widths (1–10 mm). (F) SD curves both fiber types with typical ultrasound frequency, pressure distribution and sonophore parameters, for varying fiber diameters within the physiological range of each population. (G) SD curves of equivalent “node” models of both fiber types with typical sonophore parameters and pressure distributions for varying ultrasound frequencies (20 kHz–4 MHz). (H) SD curves of “node” models with typical pressure distribution, ultrasound frequency and sonophore coverage fraction for varying sonophore radii (16–64 nm). (I) SD curves of “node” models with typical pressure distribution, ultrasound frequency and sonophore radius, for varying sonophore coverage fractions (50–100%).

First, we evaluated the excitability of representative myelinated and unmyelinated axons (10 μm and 0.8 μm diameters, respectively) with a typical stimulus width (5 mm). With electrical stimulation, threshold peak extracellular voltages required to elicit a traveling AP decreased with increasing pulse duration, and then reached an asymptotic (so-called “rheobase”) regime for long enough pulses (Figure 4A). In line with previous modeling studies (Lubba et al., 2019; Tarnaud et al., 2018), excitation thresholds for the myelinated axon were lower than those of the unmyelinated axon over the entire range of pulse durations. This result can be explained by two main factors. For short pulses where the speed of the depolarization predominantly determines when/if the spiking threshold is reached, myelinated axons can be recruited because of short membrane time constants, whereas unmyelinated axons fail to respond fast enough. Conversely, for long pulses approaching the rheobase regime, transient features become less critical and longitudinal gradients of the applied extracellular voltage become the main determinant of axonal excitability (Warman et al., 1992). Here again, myelinated axons are easier to recruit because of their insulated internodes that effectively discretize the voltage field at sparsely distributed Ranvier nodes, thereby producing stronger longitudinal gradients at the central node than those encountered across the continuous membrane of unmyelinated axons. With ultrasonic stimulation, threshold peak acoustic pressure amplitudes required for excitation also decreased with increasing pulse durations and reached a rheobase regime for long enough pulses (Figure 4D). Similarly as with electrical stimulation, short membrane and axial time constants conferred a low response latency to myelinated axons (see Figure 3), thereby allowing excitation by short ultrasonic pulses to which unmyelinated axons failed to respond. Surprisingly, however, for longer pulse durations (PD ≥ 10 ms), the SONIC paradigm predicted lower excitation thresholds in unmyelinated axons than in myelinated axons.

Second, we characterized the impact of stimulus beam width and fiber diameter on excitability by systematically exploring relevant parameter ranges and using the “chronaxie point” (i.e., the pulse duration at which the threshold is twice the rheobase, see Figures 4A and 4D) as a reference point to measure the rigid translation of SD curves in the (pulse duration – stimulus amplitude) space. With electrical stimulation, narrowing stimulus beam widths enhanced excitability of myelinated and unmyelinated axons by producing stronger longitudinal gradients in extracellular voltage (Figure 4B). These stronger gradients primarily translated SD curves toward lower thresholds, but they also slightly diminished chronaxie durations. Very narrow beams produced an inversion of rheobase values, and unmyelinated axons became easier to recruit with long enough pulses. In a mirroring manner, increasing fiber diameters also enhanced excitability in both axon types (Figure 4C) as a result of (i) a larger intracellular conductance amplifying depolarization in response to a given extracellular voltage gradient, and (ii) in myelinated axons, an increased internodal spacing (*L* = 100*D*_fiber_) that further amplifies longitudinal gradients between consecutive Ranvier nodes. Again, both of these effects induced considerable shifts of SD curves toward lower thresholds and slightly reduced chronaxie durations in both axon types. Conversely, with ultrasonic stimulation, SD curves were remarkably consistent across a range of stimulus beam widths, as well as across the physiological range of fiber diameters of both populations, with only very slight variations in the chronaxie point and no clear trend emerging (Figures 4E and 4F).

The relatively low sensitivity of ultrasonic excitation thresholds to stimulus beam width and fiber diameter suggest that excitability patterns are primarily dictated by the magnitude of the peak acoustic pressure along the axon, rather than by the beam shape or the axial properties of the axon. To verify that hypothesis, we carried out the same excitability analysis in point-neuron models representing isolated neuronal compartments of the two axon models, namely a SENN Ranvier node and a Sundt unmyelinated segment, referred to as “node” models. We found almost identical SD curves between the node and full axon models (Figure 4D), thereby confirming that excitation is primarily mediated by the localized action of acoustic pressure on the cellular membrane. At first glance, these results seem to challenge the observation that axial currents contribute significantly to the initial charge build-up at the central node of myelinated axons upon sonication (Figure 3), and may therefore indicate the presence of a sharp transition in the mechanical response of the membrane to intensifying acoustic fields, bringing axons from passive to active responses within narrow amplitude ranges. Nevertheless, these results suggest that LIFUS-triggered excitation is primarily a local phenomenon – at least in these models – that can be accurately predicted without considering extended morphological details.

Given the high accuracy of node models in predicting cell-type-specific excitation thresholds, we leveraged their computational efficiency to explore the impact of acoustic frequency, sonophore size and sonophore coverage on neuronal excitability. In line with previous modeling results in CNS neurons (Lemaire et al., 2019; Plaksin et al., 2014) we found that ultrasound frequency does not significantly affect excitation thresholds apart from a slight increase above 1 MHz due to higher viscous stresses limiting sonophore cavitation (Figure 4G). Moreover, increasing sonophore radii induced mainly a “horizontal” shift of excitability toward shorter durations (Figure 4H), while increasing sonophore coverage fractions reduced both threshold baselines and chronaxie durations (Figure 4I). Importantly, neither of these important parameter variations shifted the relative recruitment orders between myelinated and unmyelinated fibers.

### Fiber-specific rheobase excitability stems from differences in membrane capacitance

Strength-duration analyses revealed that unmyelinated axons exhibited lower excitation thresholds for long ultrasonic pulses, a trend robust to variations in model and stimulus parameters. Thus, we aimed to investigate the underlying mechanisms supporting this enhanced excitability using cell-type-specific node models, which proved to be appropriate benchmark tools to study ultrasonic neuronal recruitment (Figure 4).

**Figure 5:**
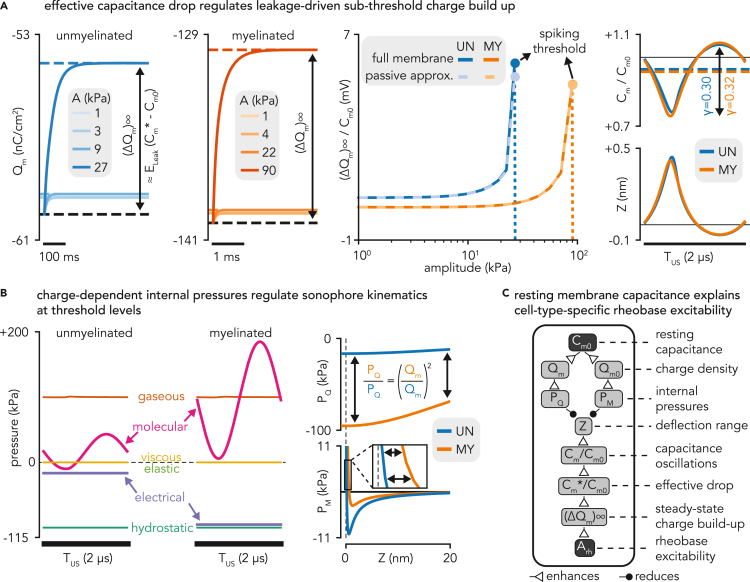
Underlying mechanisms of distinct rheobase excitabilities in myelinated and unmyelinated axons (A) Effective capacitance variations regulate sub-threshold charge build-ups. From left to right: LIFUS-triggered, exponentially converging charge build-ups in myelinated and unmyelinated “node” models for various sub-threshold pressure phasor amplitudes. Normalized steady-state charge build-ups for each “node” model as a function of sub-threshold pressure phasor amplitude, computed from full membrane simulations (plain lines) and estimated from the sole relative variation in effective membrane capacitance (dashed lines, passive circuit approximation). Detailed intra-cycle oscillation profiles of membrane capacitance and membrane deflection for each fiber type at their respective threshold levels. (B) Charge-dependent electrical and molecular pressure regulate threshold sonophore kinematics. From left to right: detailed profiles of internal pressure forces regulating sonophore cavitation during an acoustic period, driven by cell-type-specific threshold acoustic pressures. Detailed profiles of electrical and molecular pressures in both fiber types along the physiological range of membrane deflection. (C) Schematic diagram showing the causal chain of influence by which resting membrane capacitance affects charge-dependent internal pressures, sonophore kinematics, effective capacitance variations, and ultimately rheobase excitability.

For sub-threshold acoustic amplitudes and rheobase pulse durations, both the myelinated and the unmyelinated nodes responded to sonication with a build-up in charge density toward a more depolarized steady-state (Figure 5A). Increasing the acoustic amplitude enhanced the magnitude of this build-up until the node’s spiking threshold was reached and an AP was fired. Interestingly, the exponential convergence of sub-threshold charge build-ups indicated that they were mostly mediated by passive currents, and could therefore be approximated by a simple RC membrane circuit with a single leakage conductance. Under this approximation, the steady-state charge build-up is proportional to the variation of effective membrane capacitance $\left(C_{m}^{*}={\left[1/T_{US}\int_{t}^{t+T_{US}}dt/C_{m}\left(Z(t)\right)\right]}^{-1}\right)$ from its resting value:

(Equation 1) $$\begin{array}{l} {\left(\frac{dQ_{m}}{dt}\right)}_{sub-threshold}\approx -g_{Leak}\left(V_{m}^{*}-E_{Leak}\right)\\ {\left(\text{Δ}Q_{m}\right)}_{\infty }\approx \left(C_{m}^{*}-C_{m0}\right)E_{leak} \end{array}$$

For both cell types, the passive circuit approximation can accurately predict the magnitude of steady-state charge build-up across a cell-type-specific range of sub-threshold acoustic amplitudes. This high prediction accuracy confirms that sub-threshold dynamics are almost entirely governed by the drop in effective capacitance.

Both the myelinated and unmyelinated nodes required similar normalized charge build-ups to reach the spiking threshold (5.0 and 5.9 mV, respectively), which corresponded to comparable relative variations in effective membrane capacitance (−6.3 and −5.2%). Moreover, looking at intra-cycle dynamics, the resemblance of these effective cycle-averaged values arose from analogous oscillation profiles of membrane capacitance over an acoustic period (normalized oscillation ranges of 0.32 and 0.30, respectively). Recalling that capacitance is defined here as a deflection-dependent variable (see STAR Methods), this cross-model analogy could be mapped further back to cavitation profiles. Surprisingly, however, these similar membrane deflections were achieved at significantly different acoustic pressure phasor amplitudes (91 kPa and 28 kPa, respectively). This discrepancy indicates variations in the internal kinetic system regulating sonophore cavitation dynamics in each node model.

Closer inspection of the detailed oscillation profile and resulting signal energy of each internal pressure component at these cell-type-specific amplitudes revealed several interesting features (Figure 5B). First, the relatively small cavitation magnitudes and velocities for these threshold levels $\left(\left|Z\right|<0.5\;\text{nm},\left|\frac{dZ}{dt}\right|<1\;\text{cm}/\text{s}\right)$ did not generate significant viscoelastic stresses on the membrane and surrounding medium. Moreover, this cavitation dynamics allowed for an instantaneous equilibration of gaseous and hydrostatic pressures on both sides of the sonophore cavity through transmembrane gas transport, thereby yielding identical energy levels for these pressure components across the two models. In contrast, both electrical and molecular pressures showed much larger energy levels for the myelinated sonophore model than for its unmyelinated counterpart. More specifically, the molecular pressure profile was shifted toward more positive values and showed higher oscillation amplitudes, whereas the electric pressure profile was constant across a cycle but shifted toward more negative values. Together, these two pressure components are responsible for the cell-type-specificity of sonophore cavitation kinetics.

These changes in dynamic pressure oscillations can be mapped back to distinct profiles over a reference range of membrane deflections, allowing for the elucidation of the mechanisms of cell-type-specific rheobase excitability:

• The electric pressure accounts for the attraction forces between the electric ion charges on the membrane leaflets, and is defined as $P_{Q}\left(Z,\;Q_{m}\right)=-\frac{S_{0}}{S\left(Z\right)}\frac{Q_{m}^{2}}{2\varepsilon_{0}\cdot \varepsilon_{r}}$. Therefore, both electric pressure profiles show a weak dependence on membrane deflection, and a constant magnitude ratio across the deflection range $\left(\frac{P_{Q,myel}}{P_{Q,unmyel}}=5.8\right)$, corresponding exactly to the square of the ratio of threshold charge densities across the two models $\left(\frac{Q_{thr,myel}}{Q_{thr,unmyel}}=2.4\right)$. The latter ratio primarily arises from variations in a fundamental biophysical property: the resting specific membrane capacitance of the myelinated axon is twice as high as that of the unmyelinated axon ($C_{m0}^{SENN}$ = 2 μF/cm^2^, $C_{m0}^{Sundt}$ = 1 μF/cm^2^). This increased capacitance allows the myelinated membrane to accumulate twice as much charges for identical transmembrane voltages, thereby increasing the electric pressure on the membrane and hindering sonophore expansion around threshold levels.
• The intermolecular pressure is defined by a Lennard-Jones expression integrated across the sonophore surface: $P_{M}\left(Z\right)=\frac{1}{S\left(Z\right)}\overset{2\pi }{\underset{0}{\int }}\overset{a}{\underset{0}{\int }}A_{r}\cdot \left(\gamma^{x}\;–\gamma^{y}\right)\;drd\theta$ with $\gamma =\frac{\Delta^{*}}{2z\left(r\right)+\Delta \left(Q_{m0}\right)}$. All parameters of this expression are fixed except for $\Delta^{*}$, the gap between the two membrane leaflets in the absence of charges. This parameter is calculated from a model-specific equilibrium state that depends on resting charge density, and therefore shows cell-type-specificity: the more negative resting charge density of the myelinated axon – mainly resulting from its larger capacitance – results in a smaller computed gap compared to the unmyelinated axon (1.1 nm vs 1.3 nm, respectively). Slight changes in this key parameter have profound implications on the resulting molecular pressure profiles: the smaller gap in the myelinated model reduces the amplitude of the negative (i.e., attractive) peak, and more importantly, shifts the transition toward positive (i.e., repulsive) pressure to a more positive deflection value, thereby producing much larger values of repulsive intermolecular pressure and hindering sonophore compression during an acoustic cycle around threshold levels.

The resting membrane capacitance is thus a crucial parameter that indirectly regulates the rheobase excitability of peripheral axons. This regulation is explained by a causal chain of influence (Figure 5C), can be summarized as follows: the resting capacitance influences both the resting value and the variation range of membrane charge density, thereby influencing charge-dependent internal pressures. That is, with larger capacitance, electric pressure becomes more constraining during expansion phases and intermolecular pressure becomes more repulsive during compression phases. Together, these two pressure amplifications restrict the cavitation dynamics, and thus require higher acoustic pressures to attain similar membrane deflection and resulting relative capacitance oscillation ranges. In terms of cycle-averaged dynamics, higher pressures are needed to reach a given relative drop in effective capacitance, which almost entirely regulates the sub-threshold charge build-up. Given that both axon models require similar relative charge build-ups to reach their spiking threshold, rheobase excitability is then predominantly determined by the electrical modulation of cavitation dynamics, and hence by the resting membrane capacitance. In light of this mechanism, the enhanced excitability of unmyelinated axons for long pulse durations is explained by their smaller resting capacitance.

### Pulsed LIFUS robustly modulates fiber spiking activity over time

Beyond single spike elicitation by an isolated pulse, the potential of LIFUS for neuromodulation relies on its ability to induce robust patterns of spiking activity over time. To investigate that aspect within our theoretical framework, we simulated full axon models (using the standard model parameters defined in previous sections) upon the application of 10 consecutive sonication pulses (setting the stimulus beam width to one-fifth of the fiber length), detected propagated APs on membrane charge density traces of axon extremities, and computed the resulting firing rate as the reciprocal of the average inter-spike interval over the simulation window.

We first evaluated the impact of pulsing parameters on spiking activity for a fixed acoustic pressure distribution with a peak amplitude of 300 kPa (a value falling safely above single pulse excitation thresholds of both axon models). Given the important differences in the LIFUS response time constants observed between myelinated and unmyelinated axons, we explored a relevant range of pulse durations around the axon’s single pulse chronaxie for each model.

**Figure 6:**
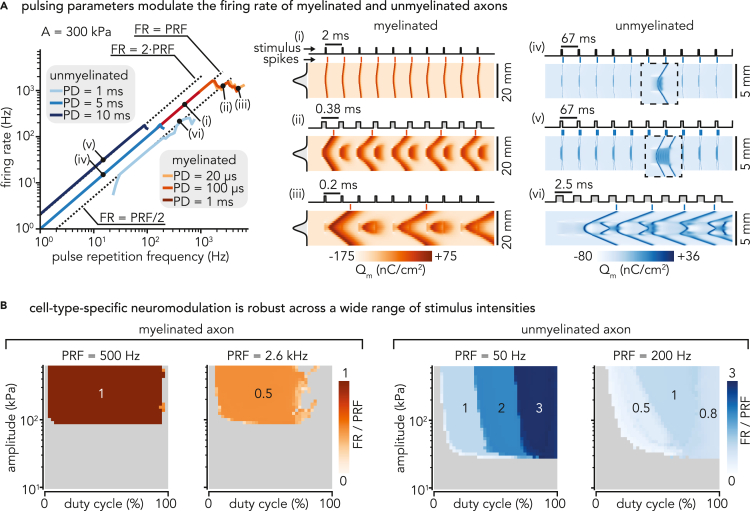
Modulation of spiking activity by pulsed sonication in myelinated and unmyelinated axons (A) Average firing rate elicited in each axon type by a Gaussian acoustic pressure distribution covering one-fifth of the fiber length, using default sonophore parameters (a = 32 nm, f_s_ = 80%) and ultrasound frequency (f_US_ = 500 kHz), for various pulse durations and pulse repetition frequencies. Dashed lines indicate half, one time and double of the stimulus rate. Detailed spatiotemporal profiles of membrane charge density are indicated for characteristic spiking regimes of each fiber type, along with detailed profiles of the stimulus spatial distribution (vertical) and temporal application (horizontal). (B) Average firing rate (normalized by pulse repetition frequency) elicited in each fiber type as a function of duty cycle and peak acoustic pressure amplitude for cell-type-specific pulse repetition frequencies yielding “robust” and “sensitive” spiking behaviors. Numbers on the color maps indicate characteristic regimes of normalized firing rate.

Myelinated axons responded with very low latency but only fired a single spike for each acoustic pulse, followed by a stabilization to a plateau potential regime. As a result, they could be driven very robustly to follow the pulse repetition frequency (PRF) up to approximately 1 kHz over a wide range of pulse durations (Figure 6A, inset (i)). At higher stimulus rates, repeated pulses started to interfere with the cell’s refractory period, thereby preventing spike generation and/or propagation on average every two pulses, causing the axon to synchronize with the half-PRF (inset (ii)). At even higher stimulus rates, only very short pulses enabled a sustained firing activity, as the axon progressively reached its physiological limit at around 1 kHz (inset (iii)).

In contrast, unmyelinated axons responded with higher latency, but could fire multiple spikes for prolonged sonication. Consequently, their behavior at low PRFs strongly depended on the pulse duration: short pulses (PD < 5 ms) did not induce any response, intermediate pulses (5 < PD < 10 ms) induced PRF-locking (inset (iv)), and long enough pulses (PD ≥ 10 ms) induced spiking activity at double or even higher multiples of the stimulus rate (inset (v)). At intermediate stimulus rates (20 Hz < PRF <100 Hz), temporal summation of sub-threshold responses enabled recruitment by short pulses at half the PRF or below. Above 100 Hz, the range of available pulse durations was progressively restricted to shorter values that only allowed the fiber to fire at half the stimulus rate or below (inset (vi)), until a physiological limit was reached around a firing rate of 200 Hz.

Having established that pulsing parameters trigger cell-type-specific patterns of spiking activity, we aimed to investigate whether these neuromodulatory effects also depend on stimulus intensity. Hence, we simulated each axon type across a two-dimensional space of duty cycles (DC, from 0 to 100%) and peak pressure amplitudes (from 10 to 600 kPa), and for each combination, computed the resulting firing rates normalized by the PRF.

At low PRFs allowing a robust pulse-spike synchronization (identified from Figure 6A for each axon type), neuromodulatory effects were surprisingly consistent across a wide range of supra-threshold stimulus amplitudes (Figure 6B). The myelinated axon fired exactly one spike per pulse for DC∈[0.02,0.95] (i.e., for pulses long enough to allow a first response yet distant enough to avoid destructive interaction with the refractory period), independently of stimulus amplitude. In contrast, the unmyelinated axon initiated a first response at slightly larger DCs and then exhibited three distinct spiking regimes with 1, 2 and 3 spikes per pulses as DC increased up to 1. A slight dependence on stimulus amplitude was noted here, as larger pressures shifted transitions between the different spiking regimes to lower duty cycles.

At high PRFs allowing only sub-stimulus rate spiking activity (see again Figure 6A), neuromodulatory effects were more intricate, and showed more dependency on stimulus amplitude. In this high frequency regime (PRF = 2.6 kHz), the myelinated axon’s firing rate approached a maximum of 0.5 times the PRF over a wide duty cycle interval (DC∈[0.04,0.70]). At larger duty cycles, spiking was only elicited for sparse DC-amplitude combinations allowing an optimal trade-off between fast depolarization to LIFUS stimuli and limited destructive interaction with the refractory period. Surrounding regions did not allow such a trade-off and could only trigger a single spike, after which the axon could not reset to fire again. In contrast, the unmyelinated axon’s spiking activity was maximized for an optimal subspace of intermediate duty cycles where the firing rate approached the stimulus rate (PRF = 200 Hz). Interestingly, larger pressures offered a wider span of this optimal DC interval. Higher duty cycles (up to 100%) also generated spiking activity but also significantly interfered with the axon’s ongoing membrane dynamics and were thus less effective.

### Multiplexed LIFUS stimuli concurrently modulate distinct fiber populations

**Figure 7:**
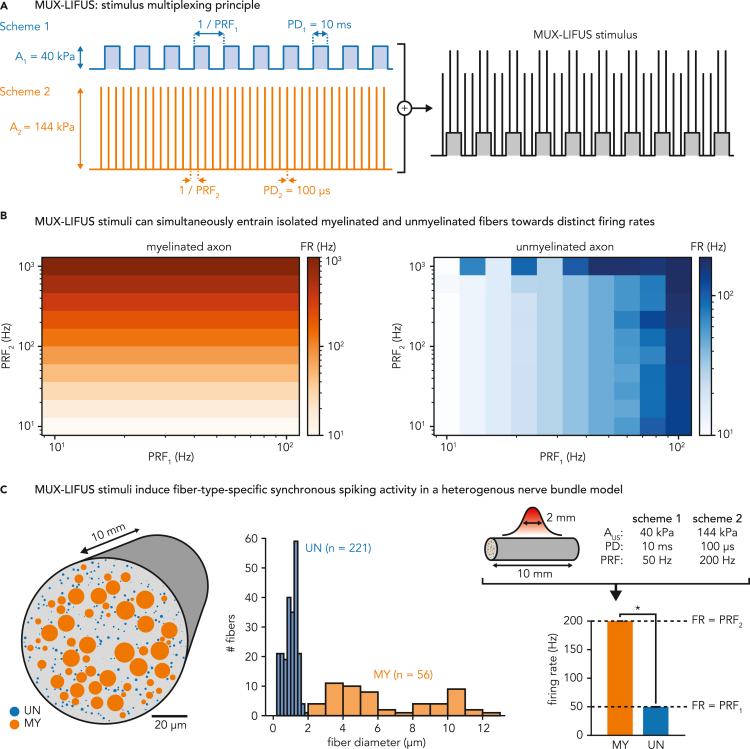
Multiplexed (MUX) LIFUS enables a robust, fiber-type-specific modulation in heterogeneous nerve populations (A) Schematic describing the LIFUS stimulus multiplexing principle. Pulse duration and pulse amplitude parameters used in the two constituent pulsing schemes are indicated. (B) Firing rates induced in representative myelinated and unmyelinated axons upon application of MUX-LIFUS stimuli, for various combinations of constituent pulse repetition frequencies (PRF_1_ = 10–100 Hz, PRF_2_ = 10–1000 Hz). Acoustic fields are modeled as a 2 mm-wide Gaussian pressure distribution along the fibers. (C) Fiber-type-specific modulation of spiking activity inside a heterogeneous nerve bundle. Left: distribution of myelinated (orange, n = 56) and unmyelinated (blue, n = 221) axons inside the bundle cross-section. Middle: total count and histogram distributions of fiber diameters of each population of the model. Right: bar plots showing the firing rates induced in each fiber population of the bundle upon application of a specific MUX-LIFUS stimulus (PRF_1_ = 50 Hz, PRF_2_ = 200 Hz). Data are represented as mean ± SD. The asterisk denotes a statistically significant difference between the two distributions, assessed using the Kruskal–Wallis test. Acoustic fields are modeled by a 2 mm-wide Gaussian pressure distribution along the bundle length, assuming uniform pressure distribution across the bundle cross-section.

Building upon the predictions of fiber-specific excitability and robust pulse-spike synchronization established in the previous sections, we investigated whether LIFUS stimuli could be engineered to independently modulate the activity of myelinated and unmyelinated fiber populations in a concurrent fashion. To this aim, we combined a short duration, high amplitude pulsing scheme (to modulate myelinated fibers) and a long duration, low amplitude scheme (to modulate unmyelinated fibers), each encoded with independent repetition rates, inside a single combined LIFUS stimulus (Figure 7A). This “multiplexed” (or “MUX”) LIFUS stimulus could then be applied to a heterogeneous population of fibers to simultaneously engage myelinated and unmyelinated fiber sub-populations toward distinct activity regimes.

We first investigated this possibility in a conceptual heterogeneous nerve model composed of only 1 fiber of each population (10 mm long, and otherwise identical to those from previous sections), exposed to a 2 mm-wide Gaussian acoustic pressure field. We defined two fundamental pulsing schemes using pulse durations tailored to target myelinated and unmyelinated fibers (100 μs and 10 ms, respectively) and pulse amplitudes 10% above the excitation threshold of the corresponding fiber population for the defined pulse durations (144 and 40 kPa, respectively). We then combined these pulsing schemes while varying their individual repetition frequency to cover a two-dimensional space, delivering at least 10 pulses of each scheme for each combination. Finally, we computed the induced spiking activity (detected by verifying spike arrival at axonal extremities) of both fibers for each applied stimulus. Simulation results showed that the MUX-LIFUS stimulus can indeed drive the spiking activity of myelinated and unmyelinated fibers (over their respective physiological range) in an independent fashion, each fiber type being uniquely sensitive to one of its constituent pulsing schemes (Figure 7B).

In light of these enticing results on single fiber models, we investigated whether our predictions could translate to the level of heterogeneous nerve fiber populations, a more relevant setting for peripheral neuromodulation applications. To this aim, we built a model of a 100 μm thick, 10 mm long cylindrical section of mixed sensory nerve bundle composed of myelinated (Aβ and Aδ) and unmyelinated (C) fiber populations realistically mimicking morphological characteristics of the human sural nerve (Jacobs and Love, 1985). We then simulated the bundle response upon exposure to a simple acoustic pressure field (2 mm-wide Gaussian distribution along the bundle length, uniform across the bundle cross-section), temporally modulated by a specific multiplexed pulsing protocol (PRF_1_ = 50 Hz, PRF_2_ = 200 Hz) (Figure 7C). Finally, we extended our spiking analysis pipeline to each constituent fiber, detecting traveling action potentials at axonal extremities and computing the resulting LIFUS-induced spiking activity. Simulation results revealed that the MUX-LIFUS stimulus application produced two clearly separated firing rate distributions for each fiber population, each synchronized with a single constituent pulsing scheme (FR_MY_ = 200.02 ± 0.01 Hz, FR_UN_ = 49.75 ± 0.26 Hz), resulting in a statistically significant difference (Kruskal-Wallis test, p < 0.001) between distributions. These results suggest that MUX-LIFUS protocols are capable of simultaneously, yet independently modulating the activity of myelinated and unmyelinated fiber populations within a heterogeneous nerve bundle. Moreover, the negligible intra-population variability in firing rate distributions emphasized the synchronicity of elicited spiking activity within each fiber group, in spite of variations in fiber diameter and relative positioning.

## Discussion

In this study, we used a novel computational framework to formulate several important predictions on the effects and mechanisms of ultrasound neuromodulation by intramembrane cavitation in peripheral fibers.

First, we predict that single LIFUS pulses are capable of inducing de novo action potentials in both myelinated and unmyelinated peripheral axons, through a common recruitment mechanism: the stimulus onset induces a local drop in effective membrane capacitance at the acoustic focus and triggers passive depolarizing currents that raise charge density toward the spiking threshold.

Second, although the two fiber types show a robust excitability across a wide range of fiber diameters, carrier frequencies and acoustic pressure fields, they exhibit distinct sensitivities to temporal features of ultrasound stimuli. Myelinated axons exhibit a low (sub-millisecond) response latency and can therefore be excited by very short LIFUS pulses for which unmyelinated axons are unresponsive. However, for longer stimuli, unmyelinated axons can be excited at lower acoustic intensities than myelinated axons. Interestingly, these distinct sensitivities are not caused by the presence/absence of myelin, but are rather attributable to specific differences in membrane biophysical properties. On the one hand, the enhanced excitability of myelinated axons for short pulse durations stems from the clustering of ion channels at the nodes of Ranvier (Kanda et al., 2019; Ritchie and Rogart, 1977; Zakon, 2012), resulting in a strong nodal membrane conductance and therefore a short membrane time constant. On the other hand, the enhanced excitability of unmyelinated fibers in the rheobase regime is attributable to a smaller specific membrane capacitance ($C_{m0}^{SENN}$ = 2 μF/cm^2^, $C_{m0}^{Sundt}$ = 1 μF/cm^2^). To the best of our knowledge, the biological origin of this capacitance difference remains undocumented. However, we found the magnitude ratio of this specific parameter to be conserved across a wide collection of biophysical models of myelinated and unmyelinated axons (Frankenhaeuser and Huxley, 1964; McIntyre et al., 2002; Tarnaud et al., 2018), which supports the reliability of our conclusions. Importantly, these differences in biophysical properties produce robust trends of fiber-specific recruitment that are hardly affected by relative changes in other loosely-constrained model parameters, such as the sonophore dimensions and density (Figures 4H and 4I). Hence, while sonophore expression levels may vary across fiber types, it should not preclude the existence of fiber-specific recruitment subspaces.

Third, the application of repeated LIFUS pulses induces a sustained spiking activity in both fiber types, the rate of which can be modulated by adjusting pulsing parameters. Particularly, myelinated axons robustly follow the stimulus rate over a wide range of PRFs, pulse durations, and supra-threshold stimulus amplitudes, while unmyelinated axons show more complex dependencies on pulse durations/duty cycle and acoustic intensities. Moreover, both fiber types can be entrained into firing rates that are comparable to those resulting from electrical stimulation (Krauthamer and Crosheck, 2002), with myelinated axons showing a much higher upper limit (FR > 1 kHz) than their unmyelinated counterparts (FR < 180 Hz). The latter finding must be interpreted with caution, as the SENN myelinated axon model ignores subtle spiking adaptation phenomena and hence probably overestimates the physiological limit of the myelinated axon’s firing rate. Importantly, robust neuromodulatory effects can be obtained with both fiber types at relatively low duty cycles (DC < 50%) that prevent significant tissue heating.

Finally, fiber-type specific LIFUS sensitivities in the two-dimensional pulse duration – pulse amplitude space can be exploited to design multiplexed LIFUS protocols that enable the concurrent, yet independent modulation of myelinated and unmyelinated fiber populations within a spatially-constrained anatomical environment such as thin sensory nerve bundles.

Together, these predictions define a comprehensive theoretical basis that can guide the design of ultrasound neuromodulation protocols.

### Applicability of the SONIC paradigm in multi-compartment models

The SONIC paradigm relies on the assumption that membrane charge density and ion channel kinetics evolve at a much slower speed than microsecond-scale capacitance oscillations, thereby allowing for the accurate integration of neural responses using precomputed cycle-averaged quantities of fast-oscillating variables. Although that assumption is valid for point-neuron models (Lemaire et al., 2019), two recent studies using a nanoscale two-compartment model has shown that under tight axial coupling conditions, strong intracellular currents mediate a significant intra-cycle charge redistribution that influences local membrane dynamics in a way that is not captured by the SONIC paradigm, resulting in overestimated sub-threshold charge build-ups and underestimated excitation thresholds (Tarnaud et al., 2020, 2021). It was also demonstrated that this numerical inaccuracy could be resolved by taking into account a limited number of Fourier components from precomputed oscillatory variables (as opposed to the SONIC approach that only considers their first component). Those findings raise legitimate concerns about the applicability of the SONIC paradigm in multi-compartment models and prompted us to examine the conditions of its applicability, and whether it can be accurately used with the axon models of this study.

First, using a generic passive benchmark, we showed that SONIC accuracy is impacted by both intrinsic model properties and stimulus features, but also that this paradigm shows robust convergence if the underlying (membrane and axial) time constants of the considered neuron model are longer than the stimulus periodicity. Second, using axon-specific benchmarks, we demonstrated that the SONIC paradigm can accurately compute passive and active neural responses of both axon models of this study, across a vast majority of the LIFUS parameter space.

In the case of the unmyelinated axon, the axial time constant is a direct product of the spatial discretization of a continuous membrane ($\tau_{ax}=C_{m0}\rho_{a}\frac{L^{2}}{D}$, with *L* the compartment length). Hence, for small enough compartments, this time constant may become smaller than the stimulus periodicity and therefore sensitize the electrical system to intra-cycle variations. However, increasing the model resolution would also eliminate the spatial gradient in acoustic pressure across consecutive compartments, effectively eradicating the axial currents at the origin of SONIC numerical inaccuracy. In fact, the selected compartment length in this study (see STAR Methods section) is in the order of 10^−2^ mm, i.e., already two orders of magnitude smaller than millimeter-scale pressure field variations.

Whether SONIC convergence can extend to other morphological models remains an open question, in particular as neurons of the CNS have a much slower membrane dynamics than peripheral axons (membrane time constants in the order of tens of milliseconds (Pospischil et al., 2008)) but possess tightly connected and heterogeneous morphological sections that may induce significant axial charge redistribution. In this case, a more computationally taxing approach considering extended Fourier decomposition might be required to achieve an acceptable level of accuracy.

### Generalizability of the morphoSONIC framework

Owing to its intrinsic mechanoelectrical coupling, the NICE model is inherently tedious to simulate. In fact, capacitance oscillations induced by the mechanical membrane resonance introduce a high frequency capacitive displacement current $\left(I_{C}=V_{m}\frac{dC_{m}}{dt}\right)$ that greatly increases the associated numerical stiffness. We first observed that this stiffness could be reduced by recasting the differential system in terms of charge density (Lemaire et al., 2019). This strategy has since been employed in two other studies implementing the NICE model (Tarnaud et al., 2020, 2021), and is also at the core of the SONIC paradigm. Unfortunately, neither time-varying capacitance nor charge casting are natively supported by standard neuronal simulation environments such as NEURON. Consequently, computational studies on intramembrane cavitation have been implemented in custom software (MATLAB or Python) and restricted to single and two-compartment models, partly because sub-optimal integration routines yield exorbitant simulation times and/or numerical instabilities for larger models.

Here, we derived a hybrid (charge and voltage casted) variant of the cable equation that is numerically reconcilable with both the NICE and SONIC paradigms and implemented it as an independent module that can be seamlessly integrated within the NEURON simulation environment. As such, the proposed approach provides a general solution to the problem of time-varying capacitance that is applicable to a wide variety of model types (single and double cable) and morphological structures (compartment number, branching patterns) seen across the central and peripheral nervous systems. Notably, this approach could also be used with enriched membrane mechanisms including lookup tables for additional Fourier components, as in (Tarnaud et al., 2020). Moreover, the choice of a NEURON-based implementation offers several advantages. First, it leverages NEURON’s optimized numerical integration pipelines while offering an appreciable abstraction level to the underlying differential systems. Second, it is applicable to a wide collection of biophysical models – as well as other resources – made available by the NEURON community (McDougal et al., 2017) with limited adaptation effort. Finally, although it has been used here with Gaussian field distributions approximating analytical solutions to simple physical problems, the morphoSONIC framework can easily be combined with finite-element-method (FEM) approaches. This refined multi-scale approach would enable the coupled simulation of complex acoustic propagation, pressure field distribution, and resulting neuronal responses inside anatomically accurate inhomogeneous tissue (such as the brain or the nerve environment).

### Comparison with empirical findings

As stated before, one of the major findings of this modeling study is that short LIFUS pulses are capable of inducing de novo action potentials in both myelinated and unmyelinated peripheral axons. This modeling prediction is in agreement with experimental observations from recent studies showing that in vivo sonication of the mouse intact sciatic nerve directly activates myelinated fibers to induce motor responses (Downs et al., 2018; Kim et al., 2020; Lee et al., 2020), and that *ex vivo* sonication of unmyelinated crab leg nerve bundles generates compound action potentials (Wright et al., 2017). Interestingly, these studies reported significantly higher excitation thresholds (3.2 MPa and 24 MPa peak pressure amplitudes around the fiber location for myelinated axons, and 1.8 MPa and unmyelinated axons) than the ones predicted here. Such differences could potentially arise from the intrinsic embedding of fibers within the neural tissue, increasing viscoelastic stresses on the membrane, and therefore hindering its mechanical resonance to acoustic perturbations (Krasovitski et al., 2011), a phenomenon that was not considered here. In fact, active neural responses in the extracted crab leg nerve bundles coincided with the presence of inertial cavitation in the surrounding medium, which may indicate higher thresholds for intramembrane cavitation in this specific environment. Besides, other studies focusing on cranial nerves have reported successful behavioral responses (e.g., eyeball movements) at peak acoustic pressures below 1 MPa (Kim et al., 2012), thereby emphasizing the variability of reported excitation thresholds across neural targets. Moreover, even within similar anatomical environments (Downs et al., 2018; Lee et al., 2020), threshold peak pressure amplitudes required to excite neural structures are highly dependent on the choice of acoustic carrier frequencies and beam width, which further highlights the sensitivity of reported excitation metrics to exposure conditions (such as focus width) and the need for accurate targeting. In spite of this uncertainty, it is worth noting that across these studies, the range of reported excitation thresholds for unmyelinated fibers was lower than those for myelinated fibers. Considering that all studies employed minimal stimulus durations that fall within the fibers predicted rheobase regimes (1 ms and 8 ms for the myelinated and unmyelinated cases, respectively), the higher sensitivity of unmyelinated structures corroborates our modeling predictions. It is also worth noting that shorter response latencies were observed in myelinated fibers (Δt < 1 ms) than in unmyelinated fibers ($\bar{\Delta t}$ ≈ 3.2 ms), which is also in agreement with our findings. Finally, all 3 studies reported significant variability in sonication success rate, which departs from the deterministic nature of single fiber responses predicted by our current model. Nevertheless, the similarities in qualitative behavior between our theoretical results and these empirical observations provide a first indication that intramembrane cavitation could be a physiologically relevant ultrasound neuromodulation mechanism also in the peripheral nervous system, where significant uncertainty remains regarding the underlying biophysics of stimulation. In fact, both cavitation and acoustic radiation force have been advanced to explain LIFUS-evoked action potential generation in peripheral fibers, and support for these hypotheses relies on concurrent empirical observations (presence of extracellular cavitation during successful trials in one case, and correlation between induced nerve displacement and response likelihood in the other case) rather than causal evidence. In this context, the theoretical framework presented in this study provides a rich body of quantitative predictions that could be tested experimentally. In particular, we suggest that a thorough comparison of excitation thresholds across fiber types and diameters, within the same nerve environment and across a wide range of pulse durations and acoustic beam widths, could be conducted in order to further assess the relevance of intramembrane cavitation as a potential mechanism of LIFUS neuromodulation.

Ultimately, access to direct recordings of membrane dynamics within the acoustic focal area upon sonication could provide further insight into the underlying biophysics of LIFUS neuromodulation. A significant effort in that direction was recently conducted by Lin et al. (2019), who used a two-electrode current clamp to record the membrane voltage of crayfish axons within the LIFUS focus. Interestingly, voltage traces of LIFUS-evoked responses recorded in this study also revealed several trends with a striking degree of similarity with our modeling predictions. In particular, authors reported that sonication consistently produced a fast hyperpolarization, followed or superseded by subthreshold depolarizations or action potentials; these observations are very reminiscent of the voltage responses predicted in this work (Figure 3) and could therefore very well be explained by a LIFUS-evoked capacitance drop, thereby providing further incentive for the potential relevance of our theoretical framework.

Yet, despite the aforementioned similarities with empirical findings, the mechanism proposed in this study does not suffice to explain the entire body of observed LIFUS neuromodulatory effects on peripheral structures. In particular, our simulations only predicted LIFUS-evoked excitation and did not reveal or hint at the possibility of LIFUS-induced silencing/blocking of nerve activity, as reported in many studies (Colucci et al., 2009; Juan et al., 2014; Kim et al., 2020; Lele, 1963; Mihran et al., 1990; Takagi et al., 1960; Young and Henneman, 1961a, 1961b); further modeling investigations on the interaction of LIFUS with physiological and electrically-induced activity will be necessary to provide a more accurate perspective on the matter. Interestingly, although reported effects were different in nature, several of these studies have also observed distinct degrees of LIFUS sensitivities between myelinated and unmyelinated fibers (with C-fibers being the most sensitive). Finally, the modulation of neural excitability over long time scales (e.g., seconds or minutes), observed over a wide range of preparations (Downs et al., 2018; Takagi et al., 1960; Young and Henneman, 1961b), is currently not captured by our modeling framework.

### Therapeutic implications

Beyond mechanistic investigation, our findings further emphasize the potential of LIFUS as a noninvasive neuromodulation technology and its applicability to peripheral structures. In fact, we predict that LIFUS enables a robust modulation of the spiking activity of both myelinated and unmyelinated fibers, thereby warranting its use to encode sensory information or elicit motor responses. In particular, the ability to selectively target unmyelinated C-fibers, which carry pain and temperature afferent signals, ushers in the possibility to encode new types of sensory information in artificial limbs without interfering with other haptic, i.e., tactile (Petrini et al., 2019; Raspopovic et al., 2014; Valle et al., 2018) and proprioceptive (D’Anna et al., 2019), modalities. To the best of our knowledge, this feature has never been achieved with standard electrical stimulation techniques. The encoding of temperature information would be particularly interesting to enrich the sensory feedback in neuroprosthetic devices and improve user experience (Mendez et al., 2021). Conversely, the absence of clear dependency of LIFUS excitation thresholds on fiber diameter represents a disadvantage, as it excludes the possibility to discriminate across different populations of myelinated fibers and, hence, to target a specific peripheral pathway. However, it is worth noting that the models used in this study employed relatively simple axonal representations, in which dimensional variations only influence extensive model properties. Here, the incorporation of more detailed axon models in which intensive properties also exhibit a diameter-dependency (McIntyre et al., 2002) could reveal finer trends of LIFUS-excitability.

The concept of selective targeting of unmyelinated or myelinated peripheral pathways by modulating acoustic amplitude and temporal stimulation parameters has already been investigated in a previous study (Legon et al., 2012), in which acoustic stimulation was delivered on nerve endings is likely to engage neural pathways through mechanoreceptors. Complementarily, this study provides a first biophysically informed insight that an energy-dependent activation can be achieved directly on axonal structures, leveraging membrane electromechanical coupling to engage their action potential machinery. Perhaps more importantly, we also predict that intrinsic differences in fiber LIFUS sensitivity can be leveraged to design multiplexed LIFUS stimuli that incorporate distinct energy components to simultaneously yet independently modulate distinct peripheral pathways. From a therapeutic perspective, LIFUS may therefore enable the encoding of a multimodal sensory feedback within spatially confined targets through a unique stimulation modality. This is of particular relevance for small nerve fascicles with heterogeneous fiber populations (such as the sural nerve bundle modeled in Figure 7C), whose dimensions (100 μm diameter) are significantly shorter than the acoustic wavelength (ca. 3 mm in this study), and in which selective stimulation cannot be achieved by means of spatial selectivity. Moreover, contrarily to hardware intensive solutions relying on acoustic field steering (e.g. ultrasonic transducer arrays), MUX-LIFUS offers a straightforward and effective solution to achieve selective peripheral neuromodulation using conventional stimulation equipment (i.e., a single element transducer controlled by a multi-channel signal generator).

### Conclusions

In this study, we present a novel computational framework to investigate the mechanisms of ultrasound neuromodulation by intramembrane cavitation in morphologically structured neuron models, using the NEURON simulation environment. Using this framework, we predict that acoustic pressure fields can modulate the spiking activity of myelinated and unmyelinated peripheral fibers in a cell-type-specific manner. These predictions agree with recent empirical observations, and open new avenues for the use of LIFUS as a neuromodulation technology in the peripheral nervous system. Yet, closer quantitative comparison with experimental data will be necessary to further validate or reject the underlying mechanism. In future work, we plan to couple our modular framework with acoustic propagation models to formulate more detailed predictions of neural responses upon sonication by realistic acoustic sources and to inform the development of application-specific ultrasonic devices.

### Limitations of the study

This study presents a computational framework to investigate a particular candidate hypothesis of ultrasound-neuron interaction – *intramembrane cavitation* – in morphologically structured axon models. As such, we did not investigate here any alternative cellular mechanisms by which ultrasound may induce neural activity, such as ion channel mechanosensitivity (Prieto et al., 2018; Sorum et al., 2021; Yoo et al., 2020), flexoelectricity (Chen et al., 2019; Petrov, 2002) or soliton spike propagation (Heimburg and Jackson, 2005). On a similar note, we focused here on the direct influence of acoustic pressure oscillation on the neural membrane and disregarded other potentially relevant ultrasound bioeffects, e.g., related to the acoustic radiation force whose importance has been demonstrated ex vivo (Menz et al., 2019) in different exposure regimes than those investigated here. The candidate cellular mechanism considered in this work has not been directly evidenced in experimental settings. However, the NICE model has been uniquely successful in providing quantitative predictions that agree with the vast majority of available experimental data of LIFUS-evoked brain activity within the low-frequency (i.e., sub-MHz) exposure regime (Plaksin et al., 2016), and the present study suggests that it may also explain LIFUS effects observed in peripheral structures. Nevertheless, owing to a lack of experimental characterization, the NICE model entails inherent assumptions that are likely an oversimplification of reality (e.g., the assumption of circular sonophore symmetry), as well as uncertainty regarding the physiological range of its parameters (e.g., sonophore diameter and coverage fraction). This uncertainty results in a loosely constrained model parameter space, and therefore a range of possible effect magnitudes for a given stimulus, which limits the prediction accuracy of metrics such as excitation thresholds. Our study generates new predictions that can be tested experimentally to validate (or falsify) the mechanism and constrain critical model parameters. Beyond its focus on a specific subcellular mechanoelectrical transduction mechanism, this work uses a Hodgkin–Huxley formalism used to model membrane electrical dynamics, which also entails simplifying assumptions about the gating dynamics ion channels (e.g., considering uncoupled states and neglecting stochastic transitions) that could affect predictions.

The axon models used in this study also involve morphological simplifications that may limit the realism of our predictions. For instance, the SENN model omits specific features of myelinated axons (namely transmembrane internodal dynamics and extracellular longitudinal coupling), that are incorporated in other, more complex fiber models (McIntyre et al., 2002). As such, the SENN axon model does not capture subtle spiking adaptation phenomena and hence probably overestimates the physiological limit of the myelinated axon’s firing rate. Nonetheless, this model incorporates enough morphological complexity to provide quantitatively accurate predictions of myelinated fiber excitability by electric fields; it is in fact a standardized model for electromagnetic exposure safety assessment (Reilly and Diamant, 2011). Moreover, given the absence of available experimental data, this study also assumes uniform sonophore distributions across active membrane compartments that are identical in both fiber models, which is likely to be an oversimplification of reality. Although that assumption influences the ratio of excitation thresholds across fiber types, it is unlikely that the existence of fiber-specific recruitment subspaces depends on that, as discussed above.

Finally, the distributions of acoustic and electrical exposure along a fiber have been approximated here by Gaussian shapes which, although qualitatively valid in nature, do not capture the full complexity of field exposure within the physically heterogeneous mammalian macro- and micro-anatomy. However, because of their inherent simplicity, Gaussian approximations allowed us to investigate general trends about the impact of field distributions on axon excitability – a central objective of this work.

## STAR★ Methods

### Key resources table

**Table:** 

REAGENT or RESOURCE	SOURCE	IDENTIFIER
**Software and Algorithms**		

NEURON 7.5	(Hines and Carnevale, 1997)	https://www.neuron.yale.edu/neuron/
Python 3.7	Python Software Foundation	https://www.python.org/downloads/
Simulation and analysis code	PySONIC library MorphoSONIC library This paper	https://github.com/tjjlemaire/PySONIC https://github.com/tjjlemaire/MorphoSONIC https://github.com/tjjlemaire/ISCIENCE-D-21-00690

### Resource availability

#### Lead contact

Further information and requests for resources should be directed to and will be fulfilled by the lead contact, Théo Lemaire (theo.lemaire@epfl.ch).

#### Materials availability

• This study did not generate new unique materials.

### Method details

#### The neuronal intramembrane cavitation excitation (NICE) model

The NICE electromechanical model developed by (Plaksin et al., 2014) provides a mathematical formulation of the intramembrane cavitation hypothesis. Mechanically, the periodic cavitation of a single bilayer sonophore is described by two differential variables: the deflection of a leaflet apex from its resting position in the transmembrane plane (*Z*) and the internal gas content in the sonophore cavity (*n*_*g*_). The resting leaflet position results from a pressure balance between several static pressure forces, namely the elastic tension developing in the leaflets (*P*_*S*_), attractive and repulsive intermolecular forces between leaflets (*P*_*M*_), internal gas pressure in the sonophore cavity (*P*_*G*_), the electric pressure resulting from the membrane polarity (*P*_*Q*_), and a constant hydrostatic term (*P*_*0*_). Upon perturbation by a time-varying acoustic pressure *P*_*A*_*(t)*, the dynamic pressure imbalance drives a normal acceleration that deforms the leaflets in antiphase, generates viscous forces in the membrane (*P*_*VS*_) and surrounding medium (*P*_*VL*_), and triggers gas transport across the cavity. These oscillatory dynamics are captured by the following differential system (all pressure terms and parameters are defined in Lemaire et al. (2019)):

(Equation 2) $$\begin{array}{l} \frac{d^{2}Z}{dt^{2}}=\frac{-3}{2R(Z)}{\left(\frac{dZ}{dt}\right)}^{2}+\frac{1}{\rho_{l}\cdot \left|R(Z)\right|}\left[P_{A}(t)+P_{S}\left(Z\right)+P_{VS}\left(\frac{dZ}{dt}\;\right)-P_{0}+P_{VL}\left(\frac{dZ}{dt}\right)+P_{M}\left(Z\right)+P_{G}(Z,\;n_{g})+P_{Q}(Z,\;Q_{m})\right]\\ \frac{dn_{g}}{dt}=\frac{2S(Z)\cdot D_{gl}}{\xi }\left(C_{g}-\frac{P_{G}(Z)}{k_{H}}\right) \end{array}$$

Electrically, the development of an electrical response across the membrane is captured by a modified Hodgkin-Huxley differential system, describing the evolution of the membrane charge density (*Q*_*m*_) as the negative sum of voltage-dependent ionic currents with specific conductances *g*_*i*_ and reversal potentials *E*_*i*_. In this system, time-varying ionic conductances are the product of one or multiple gating variables (*x,* with *x* ∈ {*m*, *h*, *n*, *p,* …}), whose evolution is regulated either by voltage-dependent activation and inactivation rate constants (*α*_*x*_ and *β*_*x*_, respectively) or by a steady-state probability *x*_*∞*_ and a time constant *τ*_*x*_ (also both voltage-dependent), yielding the following system (note that charge-casting was introduced in Lemaire et al. (2019)):

(Equation 3) $$\begin{array}{l} \frac{dQ_{m}}{dt}=-\left[\;\underset{i}{\sum }g_{i}\cdot \left(\frac{Q_{m}}{C_{m}}-E_{i}\right)\right]\;\\ \frac{dx}{dt}=\left\{ \begin{array}{l} \alpha_{x}\left(\frac{Q_{m}}{C_{m}}\right)\cdot \left(1-x\right)-\beta_{x}\left(\frac{Q_{m}}{C_{m}}\right)\cdot \;x\\ \frac{x_{\infty }\left(\frac{Q_{m}}{C_{m}}\right)-x}{\tau_{x}\left(\frac{Q_{m}}{C_{m}}\right)} \end{array}\right. \end{array}$$

The coupling between these two systems is modeled by a bidirectional piezoelectric effect. Mechanoelectrical transduction results from the periodic deflections of the sonophore leaflets, inducing high frequency oscillations in the local membrane capacitance (given by $C_{m}\left(t\right)=\frac{C_{m0}\text{Δ}}{{\text{a}}^{2}}\left[Z\left(t\right)+\frac{a^{2}-Z{\left(t\right)}^{2}-Z\left(t\right)\cdot \text{Δ}}{2Z\left(t\right)}\ln\left(\frac{2Z\left(t\right)+\Delta }{\Delta }\right)\right]$, as in Plaksin et al. (2014)). Considering a larger, macroscale portion of membrane area, local fluctuations of membrane capacitance around individual sonophores influence the spatial average of membrane capacitance, calculated as a weighted mean of the resting and dynamic capacitances:

(Equation 4) $$C_{m}=C_{m}\left(t\right)f_{s}+C_{m0}\left(1-f_{s}\right)$$

where *f*_*s*_ is the sonophore membrane coverage fraction). This global fluctuation then causes large amplitude oscillations of the transmembrane potential in the compartment of interest ($V_{m}=Q_{m}/C_{m}$ in Equation (3)). Reversibly, electromechanical transduction results from progressive changes in the membrane electrical polarity that dynamically modify the electric pressure exerted on the sonophore leaflets and the resulting pressure balance (*P*_*Q*_ in Equation (2)), thereby influencing the sonophore cavitation dynamics.

#### The multi-Scale Optimized Neuronal Intramembrane Cavitation (SONIC) model

The SONIC model (Lemaire et al., 2019) uses temporal multi-scaling to separate the two relevant time scales of the NICE model, namely microsecond-scale mechanical oscillations and millisecond-scale development of neuronal responses. It is based on the observation that ion channel gates – whose time constants are typically in the millisecond range – do not follow large amplitude, high frequency variations of transmembrane potential observed in the NICE model, but rather adapt to the temporal average of voltage oscillations over an acoustic cycle. As a result, the evolution of membrane charge density and ion channels gating variables can be expressed as a function of an effective membrane potential $\left(V_{m}^{*}\right)$ and effective activation and inactivation rate constants ($\alpha_{x}^{*}$ and $\beta_{x}^{*}$, respectively, for each gating variable *x*), representing the average value of their original, voltage-dependent counterparts (*V*_*m*_, *α*_*x*_ and *β*_*x*_, respectively) over an acoustic cycle:

(Equation 5) $$\begin{array}{l} \frac{dQ_{m}}{dt}=-\underset{ion}{\sum }g_{ion}\cdot \left(V_{m}^{*}-E_{ion}\right)\\ \frac{dx}{dt}=\alpha_{x}^{*}\;\cdot \left(1-x\right)-\beta_{x}^{*}\;\cdot x \end{array}$$

The SONIC model uses a sequential approach to compute electrical responses of a given neuron type to various LIFUS stimuli. First, a parallelized precomputation step is performed (once per neuron type) in which the mechanical system (Equation (2)) is simulated for various combinations of sonophore radii (*a*), ultrasound frequencies (*f*_*US*_), acoustic pressure phasor amplitudes (*A*_*US*_), and membrane charge densities, covering the LIFUS parametric space, sonophore geometrical range and membrane physiological range. Each simulation is run until a limit cycle is detected, at which point, the profile of oscillating membrane capacitance *C*_*m*_*(t)* over the last acoustic cycle is extracted, rescaled according to a specific sonophore membrane coverage fraction using Equation (4), and converted to a corresponding voltage profile $V_{m}=Q_{m}/C_{m}$. The effective membrane potential $V_{m}^{*}$ and rate constants $\alpha_{x}^{*}$ and $\beta_{x}^{*}$ (for each gating variable *x*) are then computed as:

(Equation 6) $$\begin{array}{l} V_{m}^{*}=\frac{\int_{0}^{T_{US}}V_{m}\left(t\right)dt}{T_{US}}\\ \alpha_{x}^{*}=\frac{\int_{0}^{T_{US}}\alpha_{x}\left(V_{m}\left(t\right)\right)dt}{T_{US}}\\ {\text{β}}_{x}^{*}=\frac{\int_{0}^{T_{US}}\beta_{x}\left(V_{m}\left(t\right)\right)dt}{T_{US}}\; \end{array}$$

and stored in multidimensional lookup tables. Second, the electrical response of the neuron to a given LIFUS stimulus is rapidly computed at runtime by interpolating effective variables at (*a, f*_*s*_*, f*_*US*_*, A*_*US*_) and (*a, f*_*s*_*, f*_*US*_*, 0*) to yield 1D projected vectors in the *Q*_*m*_ space, which are then used to interpolate effective variables and solve Equation (5) during LIFUS-ON and LIFUS-OFF periods, respectively.

#### A hybrid multi-compartment, multi-layer circuit

In its most basic form, the multi-compartment expansion of point-neuron NICE/SONIC models requires the addition of axial current terms contributing to the evolution of charge density in each compartment (see (Lemaire et al., 2019), Equation 5). However, that formulation only considers intracellular axial coupling, and is therefore not adapted to double-cable models that account for both intra and extracellular longitudinal coupling. More importantly, the use of explicit current terms representing axial coupling is prone to yielding numerical instabilities in the presence of tightly connected sections or abrupt changes in voltage gradients. Hence, in this study, we derived a hybrid multi-compartment multi-layer electrical circuit that is applicable to both single and double cable structures with temporally and spatially varying membrane capacitances, and compatible with reference numerical integration schemes and simulation environments.

The circuit model is composed of multiple longitudinal compartments, each represented by a pair of intracellular and extracellular voltage nodes (*V*_*i*_ and *V*_*x*_, respectively) on either side of the plasma membrane with time-varying capacitance *C*_*m*_*(t)*. The voltage difference across the plasma membrane $V_{m}^{*}$ = *V*_*i*_ - *V*_*x*_ influences the opening and closing of distinct ion channels, ultimately giving rise to a net membrane ionic current *I*_*ion*_. On the extracellular side, a transverse resistor-capacitor (RC) circuit of conductance *g*_*x*_ and capacitance *C*_*x*_ represents the myelin membrane and connects the extracellular node to the extracellular driving voltage *Φ*_*e*_, which is usually grounded but can also have a value imposed by an external electric field. Longitudinally, neighboring nodes are connected intracellularly and extracellularly by axial conductors (*G*_*a*_ and *G*_*p*_, respectively). All variables and parameters of the circuit are described in Table 1, and a schematics of the circuit model is given in Figure S2A.

**Table 1:** Parameters and variables of the hybrid multi-compartment, multi-layer electrical circuit

Parameter/variable	Symbol	Unit
Intracellular voltage	*V*_*i*_	mV
Extracellular voltage	*V*_*x*_	mV
Transmembrane voltage	*V*_*m*_	mV
Transmembrane charge density	*Q*_*m*_	nC/cm^2^
Extracellular driving voltage	*Φ*_*e*_	mV
Membrane capacitance	*C*_*m*_	μF/cm^2^
Intracellular stimulation current	*I*_*s*_	mA/cm^2^
Net transmembrane ionic current	*I*_*ion*_	mA/cm^2^
Intracellular axial conductance	*G*_*a*_	S
Capacitance of surrounding extracellular membrane (e.g., myelin)	*C*_*x*_	μF/cm^2^
Transverse conductance of surrounding extracellular membrane (e.g., myelin)	*g*_*x*_	S/cm^2^
Extracellular axial conductance (e.g., periaxonal space)	*G*_*p*_	S
Membrane area of the compartment	*A*_*m*_	cm^2^

For any compartment *k* connected to a set of neighboring compartments, the application of Kirchhoff’s law at the corresponding intracellular and extracellular nodes yields the following current balance equations:

(Equation 7) $$\begin{array}{l} C_{m}^{k}\frac{dV_{m}^{k}}{dt}+V_{m}^{k}\frac{dC_{m}^{k}}{dt}\;+\;I_{ion}^{k}\;=\;I_{s}^{k}\;+\;\frac{1}{A_{m}^{k}}\underset{intracellular\;axial\;current}{\underset{︸}{\underset{j}{\sum }G_{a}^{kj}\;\left(V_{i}^{j}\;-\;V_{i}^{k}\right)}}\\ C_{x}^{k}\frac{dV_{x}^{k}}{dt}\;+\;g_{x}^{k}\;\left(V_{x}^{k}\;-\;\varphi_{e}^{k}\right)=\;C_{m}^{k}\frac{dV_{m}^{k}}{dt}\;+\;I_{ion}^{k}\;+\frac{1}{A_{m}^{k}}\underset{periaxonal\;axial\;current}{\underset{︸}{\underset{j}{\sum }G_{p}^{kj}\;\left(V_{x}^{j}\;-\;V_{x}^{k}\right)}} \end{array}$$

Using $V_{i}\;=V_{m}\;+\;V_{x}$, and re-arranging the terms, we find:

(Equation 8) $$\begin{array}{l} C_{m}^{k}\frac{dV_{m}^{k}}{dt}+V_{m}^{k}\frac{dC_{m}^{k}}{dt}+\frac{1}{A_{m}^{k}}\underset{j}{\sum }G_{a}^{kj}\;\left(V_{m}^{k}\;-\;V_{m}^{j}\right)+\frac{1}{A_{m}^{k}}\underset{j}{\sum }G_{a}^{kj}\;\left(V_{x}^{k}\;-\;V_{x}^{j}\right)\;=\;I_{s}^{k}-I_{ion}^{k}\\ C_{x}^{k}\frac{dV_{x}^{k}}{dt}-\left(C_{m}^{k}\frac{dV_{m}^{k}}{dt}+V_{m}^{k}\frac{dC_{m}^{k}}{dt}\right)+g_{x}^{k}\;V_{x}^{k}+\frac{1}{A_{m}^{k}}\underset{j}{\sum }G_{p}^{kj}\;\left(V_{x}^{k}\;-\;V_{x}^{j}\right)=\;I_{ion}^{k}\;+g_{x}^{k}\varphi_{e}^{k} \end{array}$$

By substituting transmembrane voltage for transmembrane charge density $\left(Q_{m}\left(t\right)=C_{m}\left(t\right)\cdot V_{m}\left(t\right);\;\frac{dQ_{m}}{dt}=C_{m}\frac{dV_{m}}{dt}+V_{m}\frac{dC_{m}}{dt}\right)$, and defining $I_{e}^{k}=\;g_{x}^{k}\varphi_{e}^{k}$ as the extracellular driving current, we obtain:

(Equation 9) $$\begin{array}{l} \frac{dQ_{m}^{k}}{dt}+\frac{1}{A_{m}^{k}}\underset{j}{\sum }G_{a}^{kj}\;\left(\frac{Q_{m}^{k}}{C_{m}^{k}}\;-\frac{Q_{m}^{j}}{C_{m}^{j}}\right)+\frac{1}{A_{m}^{k}}\underset{j}{\sum }G_{a}^{kj}\;\left(V_{x}^{k}\;-\;V_{x}^{j}\right)\;=\;I_{s}^{k}-I_{ion}^{k}\\ C_{x}^{k}\frac{dV_{x}^{k}}{dt}-\frac{dQ_{m}^{k}}{dt}+g_{x}^{k}\;V_{x}^{k}+\frac{1}{A_{m}^{k}}\underset{j}{\sum }G_{p}^{kj}\;\left(V_{x}^{k}\;-\;V_{x}^{j}\right)=\;I_{ion}^{k}\;+I_{e}^{k} \end{array}$$

By applying the above equations to a model of *n* compartments connected in series, we obtain a hybrid charge-voltage partial differential equation system of size *2n* that can be described as:

(Equation 10) $$C\frac{dy}{dt}\;+\;G\left(t\right)\cdot y\left(t\right)=\;I\left(t\right)$$

where:

• *y* is a hybrid vector of transmembrane charge density and extracellular voltage, and *dy/dt* its temporal derivative;
• *C* is a constant matrix composed of both capacitance terms (multiplying voltage elements of *dy/dt*) and “identity” terms (multiplying charge elements of *dy/dt*);
• *G(t)* is a time-varying matrix composed of both conductance terms (multiplying voltage elements of *y*) and “frequency” terms (conductance by capacitance ratios in MHz, multiplying charge elements of *y*); and
• *I(t)* is a time-varying vector of stimulation and membrane currents

This matrix formulation allows for the use of implicit methods to solve the differential equation problem, thus providing an enhanced stability over explicit schemes.

Moreover, by mapping the first *n* elements of the *y* vector to transmembrane charge density nodes and the following *n* elements to extracellular voltage nodes, we can describe the *C*, *G* and *I* terms of the system as combinations of block matrices and vectors, i.e.:

(Equation 11) $$C=\;\left[\begin{array}{ll} \left[I_{n}\right] & 0\\ \left[-I_{n}\right] & \left[C_{x}\right] \end{array}\right],\;G\left(t\right)=\;\left[\begin{array}{ll} \left[\frac{G_{a}}{A_{m}\cdot C_{m}\left(t\right)}\right] & \left[\frac{G_{a}}{A_{m}}\right]\\ 0 & \left[g_{x}\right]+\left[\frac{G_{p}}{A_{m}}\right] \end{array}\right],\;I\left(t\right)=\left[\begin{array}{l} \vec{I_{s}\left(t\right)}-\vec{I_{ion}\left(t\right)}\\ \vec{I_{ion}\left(t\right)}+\vec{I_{e}\left(t\right)} \end{array}\right],$$

where:

• $\left[I_{n}\right]$ is an n-by-n identity matrix;
• $\left[C_{x}\right]$ is an n-by-n diagonal matrix of transverse extracellular membrane (e.g. myelin) capacitance;
• $\left[\frac{G_{a}}{A_{m}}\right]$ and $\left[\frac{G_{p}}{A_{m}}\right]$ are n-by-n tridiagonal matrices of intracellular and extracellular axial conductance, respectively, where each row is normalized by the corresponding node’s membrane area;
• $\left[\frac{G_{a}}{A_{m}\cdot C_{m}\left(t\right)}\right]$ is an n-by-n tridiagonal matrix of intracellular axial conductance where each row is normalized by corresponding node’s membrane area and each column is dynamically normalized by the time-varying membrane capacitance of the corresponding node;
• $\left[g_{x}\right]$ is an n-by-n diagonal matrix of transverse extracellular membrane (e.g. myelin) conductance; and
• $\vec{I_{s}\left(t\right)}$, $\vec{I_{ion}\left(t\right)}$ and $\vec{I_{e}\left(t\right)}$ are n-sized, time-varying vectors of intracellular stimulation currents, transmembrane ionic currents and extracellular driving currents, respectively.

We implemented this hybrid system in NEURON (Hines and Carnevale, 1997), a reference computational environment for neuronal simulations that uses a very similar matrix formulation to enable numerical integration by implicit schemes. However, since that environment is not designed for models of varying capacitance or for hybrid charge-voltage casting, we employed three main adaptation strategies. First, a unit capacitance was set to all membrane mechanisms, thereby implicitly setting the $\left[I_{n}\right]$ upper block matrix and effectively transforming NEURON’s internal variable v as an alias to transmembrane charge density. Second, pressure phasor amplitude and charge density dependent lookup tables of effective SONIC terms (transmembrane potential and ion channels rate constants obtained from original SONIC lookup tables (Lemaire et al., 2019)) were dynamically inserted into these mechanisms to compute the evolution of voltage, ion channels states and ionic currents via bilinear interpolation (thereby implicitly setting the $\vec{I_{ion}}$ upper block vector). Third, alternative *C*′ *G*′ and *I*′ terms were defined to complete the hybrid circuit setup upon definition of the model’s compartments and their connections:

(Equation 12) $$C^{\prime }=\;\left[\begin{array}{ll} 0 & 0\\ 0 & \left[C_{x}\right] \end{array}\right],\;G^{\prime }\left(t\right)=\;\left[\begin{array}{ll} \left[\frac{G_{a}}{A_{m}\cdot C_{m}\left(t\right)}\right] & \left[\frac{G_{a}}{A_{m}}\right]\\ \left[\frac{G_{a}}{A_{m}\cdot C_{m}\left(t\right)}\right] & \left[\frac{G_{a}}{A_{m}}\right]+\left[g_{x}\right]+\left[\frac{G_{p}}{A_{m}}\right] \end{array}\right],\;I^{\prime }\left(t\right)=\left[\begin{array}{l} 0\\ \vec{I_{e}\left(t\right)}+\vec{I_{s}\left(t\right)} \end{array}\right].$$

These terms were added to NEURON’s currents balance equations via the use of a “Linear Mechanism” (an interface object provided by the NEURON software allowing to interact directly with its internal equations). It should be noted that the terms $\left[-I_{n}\right]$ and $\vec{I_{ion}\left(t\right)}$ in the lower block are replaced by equivalent axial conduction and intracellular stimulation current terms (by adding the equality of the upper block) to remove the need to access the net membrane current, a hidden NEURON variable. Numerical integration is then carried out by NEURON’s embedded general sparse matrix solver (a differential-algebraic solver with a preconditioned Krylov method from the SUNDIALS package (Hindmarsh et al., 2005)) using a variable time step with a pure absolute error tolerance criterion ($\varepsilon =10^{-3}$), while dynamically updating *C*_*m*_-dependent terms in the *G′* matrix throughout the simulation. A detailed description of the matrix formulation and its integration into the NEURON environment is given in Figure S2C. Compared to previous approaches using explicit axial current terms (Lemaire et al., 2019), this implicit integration scheme offers increased numerical stability.

#### Morphological axon models

In order to simulate intramembrane cavitation in peripheral nerve fibers, we used the hybrid circuit described above to incorporate the SONIC paradigm inside multi-compartmental models of myelinated and unmyelinated axons (Reilly et al., 1985; Sundt et al., 2015). For both fiber types, we selected established axon models that employ a single-cable representation and thereby offer a good compromise between model complexity, numerical accuracy (see Figure 2), and morphological realism.

Our myelinated axon model was based on the spatially-extended nonlinear node (SENN) model developed by Reilly et al. (1985), which underlies a range of low-frequency exposure safety standards (prevention of undesired neurostimulation) and has been extensively used in previous studies on the excitability of myelinated nerve fibers by electrical fields for various applications, including neurostimulation of somatosensory and autonomic nerves (Gupta et al., 2020; Neufeld et al., 2016; Samoudi et al., 2017), modulation of nerve excitability by multi-frequency and high-frequency currents (Makino et al., 2020; Zhao et al., 2015), and minimization of nerve excitation during electroporation procedures (Mercadal et al., 2017). This model represents myelinated axons as a set of nodes with active membrane dynamics based on the Frankenhaeuser-Huxley equations for a Xenopus Ranvier node (Frankenhaeuser and Huxley, 1964) including fast sodium (I_Na_), delayed-rectifier potassium (I_Kd_), non-specific delayed (I_P_) and non-specific leakage (I_Leak_) currents, connected by intracellular resistors representing the myelinated internodes (Figures 1A and 1B).

Our unmyelinated axon model was based on the work of Sundt et al. (2015), which constitutes a reference computational model employed in several recent investigations on the neuromodulation of unmyelinated sensory neurons during electrical stimulation of various neural structures including dorsal root ganglions (Graham et al., 2019), autonomic nerves (Gupta et al., 2020) and spinal circuits (Squair et al., 2021), as well as in physiological studies on information integration in nociceptive terminals (Barkai et al., 2020). This model represents the continuous unmyelinated neurite as a set of nodes containing fast Sodium (I_Na_), delayed-rectifier Potassium (I_Kd_), and leakage (I_Leak_) membrane currents, also connected by intracellular resistors (Figures 1C and 1D). Notably, these axon models both use single-cable representations and therefore do not require the second, extracellular layer of coupling defined in Equations 7, 8, 9, 10, 11, and 12, which was added for the sake of generality.

The selected axon models were validated numerically by verifying specific physiological features (spike amplitude, conduction velocity, threshold excitation current for various pulse widths) against the reference literature (Reilly et al., 1985; Sundt et al., 2015), using NEURON’s native voltage-based connection scheme with constant membrane capacitance. For the unmyelinated model, a convergence study was carried out to determine the optimal spatial discretization. Unmyelinated compartments were progressively and uniformly shortened from 1 mm to 5 μm, and an optimal segment length was defined as the maximal length for which all physiological features were within 5% of their converging values (obtained for the shortest segment length). As the optimum segment length exhibited a clear dependency on fiber diameter, we performed a piecewise linear fit within the 0.5–1.5 μm range to obtain a fiber diameter-dependent formulation: $L_{opt}=\min\left(16.4\cdot D_{fiber}+9.1\;\mu m;22\;\mu m\right)$. Finally, we validated our hybrid circuit implementation by comparing direct voltage traces, as well as physiological features, to those obtained with the “native” implementation.

Membrane equations of both models were adapted to 36°C by applying a Q_10_ correction with a factor of 3 (as in Sundt et al. (2015)), and lookup tables of SONIC effective variables were generated for the membrane circuits of both models to enable their simulation upon acoustic perturbations (Figure 1E).

#### Analytical models of exposure distributions

In order to evaluate the effect of exogenous electrical and ultrasonic stimulation on isolated fibers, we modeled the propagation of both electrical and acoustic fields from a realistic remote excitation source to the target through a homogeneous intraneural medium. To this end, we considered a 3-dimensional (x,y,z) coordinate system in which the fiber was aligned on the *x* axis and centered at the origin.

For ultrasonic stimulation, we considered a single-element planar acoustic transducer with a center in the xz plane and a normal vector along the z-axis, and a homogeneous, water-like propagation medium (density ρ = 1000 kg/m^3^, speed of sound c = 1500 m/s). We modeled acoustic distribution in the xz propagation plane using the Distributed Point Source Method (DPSM) (Yanagita et al., 2009), which provides accurate approximations of the Rayleigh-Sommerfeld integral (RSI) in homogeneous medium. That is, assuming a uniform particle velocity normal to the transducer surface of amplitude $v_{0}$, the complex acoustic pressure phasor at each field point (x,z) for an acoustic frequency f can be computed as:

(Equation 13) $$P_{ac}\left(x,\;z\right)=-jf\rho v_{0}\iint_{S}\frac{e^{jk_{f}d}}{d}dS,$$

where *j* is the unit imaginary number, $k_{f}=2\pi f/c$ is the wave number, and $d=\sqrt{{\left(x-x_{dS}\right)}^{2}+{\left(z-z_{dS}\right)}^{2}+y_{dS}^{2}\;}$ is the distance between the field point and a surface element dS. We numerically approximated this integral as the sum of individual contributions of a finite set of M uniformly distributed point sources – each associated with a surface area ΔS – arranged in a concentric fashion on the transducer surface:

(Equation 14) $$P_{ac}\left(x,\;z\right)=-jf\rho v_{0}\text{Δ}S\overset{M}{\underset{i=1}{\sum }}\frac{e^{jk_{f}d_{i}}}{d_{i}}.$$

The amplitude *A* and phase *ϕ* of the complex acoustic pressure field can then be recovered as:

(Equation 15) $$\begin{array}{l} A=\|P_{ac}\|\\ \varphi =\tan^{-1}\left(P_{ac}\right) \end{array}$$

Here again, we performed a sensitivity analysis to determine the optimal density of point sources required to achieve a good prediction accuracy. Starting with a low source density (10 samples/mm^2^), the predicted pressure distribution along the central _*z*_ axis was evaluated against the corresponding closed form RSI solution ($P\left(z\right)=\rho cv_{0}\left[e^{jk_{f}z}-e^{jk_{f}\sqrt{z^{2}+r^{2}}\;}\right]$, with _*r*_ the transducer radius), and source density was increased until the variation of the root-mean-square error (RMSE) fell below a threshold value (10 kPa). We then selected the minimal value satisfying that criterion over a wide frequency range (500 kHz–5 MHz), yielding an optimal density of 217 samples/mm^2^.

Finally, we evaluated pressure distributions along the transverse _*x*_ axis at the acoustic focal distance (calculated as $z_{f}=\frac{fr^{2}}{c}-\frac{c}{4f}$) for each combination of transducer radius and ultrasound frequency.

For electrical stimulation, we considered a point source electrode located in the xz plane and an anisotropic conductivity tensor characteristic of the mammalian endoneurium (longitudinal resistivity$\;\rho_{x}=175\;\Omega \cdot \text{cm}$, transverse resistivity$\;\rho_{yz}=1211\;\Omega \cdot \text{cm}$) (Ranck and Bement, 1965). Extracellular potentials at each field point (x,z) were computed with the formula:

(Equation 16) $$\varphi_{e}\left(x,z\right)=\frac{I}{4\pi \sqrt{\frac{{\left(x_{0}-x\right)}^{2}}{\rho_{yz}^{2}}+\frac{z_{0}^{2}}{\rho_{x}\cdot \rho_{yz}}}\;},$$

where *I* is the injected current and $\left(x_{0},z_{0}\right)$ are the electrode coordinates, and equivalent sets of intracellular currents were used to simulate the influence of the extracellular electric field, as in McIntyre et al. (2002).

Note that Equations (14) and (16) provide closed-form expressions to predict the qualitative nature of ultrasonic and electric field distributions along a fiber, thereby allowing general trends about the impact of those distributions on axon excitability to be established. However, they only consider propagation within a homogeneous medium, which is a limitation.

#### Mixed sensory nerve bundle model

The heterogeneous nerve bundle was approximated as a 10 mm long cylindrical section (100 μm in diameter) populated with both myelinated and unmyelinated fibers. For each subtype, fiber diameters were sampled from realistic distributions based on morphological data from the sural branch of human sciatic nerves (Jacobs and Love, 1985). A total of 221 unmyelinated and 56 myelinated fibers were generated, so as to reflect the typical 1:4 ratio of myelinated and unmyelinated fibers in this nerve branch (Jacobs and Love, 1985) whilst providing sufficient population statistics of each subtype. Generated fibers were randomly distributed within the bundle cross-section using a simple packing algorithm (preventing fiber overlap), assigned random longitudinal shifts within the ±w interval (with w the fiber-specific node-to-node distance) to avoid alignment of central nodes of Ranvier, and then spatially extended to cover the entire bundle length. For the bundle exposure by acoustic fields, we assumed a uniform acoustic pressure distribution across the bundle cross section, since the bundle diameter (100 μm) was significantly smaller than the ultrasound wavelength (ca. 3 mm at f_US_ = 500 kHz).

### Two-compartment SONIC benchmark models

The passive benchmark model was composed of two passive compartments with identical geometries and passive membrane properties (C_m0_ = 1 μF/cm^2^, V_m0_ = E_Leak_ = −70 mV). Membrane and axial conductances were mapped to equivalent time constants:

(Equation 17) $$\begin{array}{l} \tau_{m}=\frac{C_{m0}}{g_{Leak}}\\ \tau_{ax}=C_{m0}A_{m}/G_{a} \end{array}$$

For each configuration, simulation duration was fixed to five times the longest time constant (but at least 10 acoustic periods) in order to ensure convergence of all solutions toward a steady-state.

Steady-state SONIC deviation was then computed as the maximum across compartments 1 and 2 of the absolute difference in charge density between the SONIC solution $Q_{m}^{SONIC}$ and the cycle-averaged NICE solution $\left\langle Q_{m}^{NICE}\right\rangle$:

(Equation 18) $$\varepsilon_{\infty }=\max\left({\left|{\left(Q_{m}^{SONIC}\right)}_{\infty }-{\left(\langle Q_{m}^{NICE}\rangle \right)}_{\infty }\right|}_{k},\;k\in \left\{1,\;2\right\}\right)$$

Transient SONIC deviation was evaluated after normalizing SONIC and cycle-averaged NICE solutions to the unit interval, in order to evaluate differences in transient dynamics irrespective of charge build-up magnitudes. Following this normalization step, the end of the transient phase *t*_*thr*_ was identified in each compartment as the time at which the cycle-averaged NICE profile first converged within 0.1% of the unit steady-state. The absolute difference between SONIC and cycle-averaged NICE profiles, and the difference between the cycle-averaged NICE profile and the unit steady-state, were then integrated over the [0, *t*_*thr*_] interval, and transient SONIC deviation was defined as the maximum across compartments of the ratio of these two integrals:

(Equation 19) $$\varepsilon_{\tau }=\max\left(\frac{\int_{0}^{t_{thr}}{\left|Z\left(Q_{m}^{SONIC}\right)-Z\left(\left\langle Q_{m}^{NICE}\right\rangle \right)\right|}_{k}\;dt}{\int_{0}^{t_{thr}}{\left(1-Z\left(\left\langle Q_{m}^{NICE}\right\rangle \right)\right)}_{k}\;dt},\;k\in \left\{1,\;2\right\}\right),\;\;with\;Z\left(x\right)=\frac{x-\min\left(x\right)}{\max\left(x\right)-\min\left(x\right)}$$

The denominator in Equation (19) ensures that the resulting deviation metrics can be interpreted independently of the convergence time constant. This normalized metrics is meaningful when the majority of the charge variation range is comprised in the transient phase, but falls short when the charge build-up is negligible. Therefore, conditions yielding a cycle-averaged NICE charge variation range below 1 nC/cm^2^ were excluded from the transient deviation analysis.

The axon-specific benchmarks were composed of two identical compartments with axon-specific morphological properties and full membrane dynamics of each fiber type. Physiologically relevant simulation durations known to elicit spiking activity in each model (1 ms and 10 ms for the myelinated and unmyelinated cases, respectively) were used.

For each condition, SONIC deviation was evaluated in each compartment following the gamma distance evaluation method of Low et al. (1998). It compares two functions or distributions by finding for each point in the test distribution the “closest” corresponding point in the reference distribution, considering both deviations in value (dose-difference) and arguments (distance-to-agreement; in our case, the time difference). “Closest” is defined based on a multi-dimensional Euclidean distance norm of tolerance-normalized difference contributions. I.e., the Gamma-distance between a point at time t_2_ of the test (SONIC) charge density time-series and a point at time t_2_ of the reference (cycle-averaged NICE) in compartment k is calculated as:

(Equation 20) $$\text{Γ}{\left(t_{1},\;t_{2}\right)}_{k}=\sqrt{\frac{t_{0}^{2}}{\text{Δ}t^{2}}+\frac{{\left(Q_{m}^{SONIC}\left(t_{2}\right)-\left\langle Q_{m}^{NICE}\right\rangle \left(t_{1}\right)\right)}^{2}}{\text{Δ}Q_{m}^{2}}}$$

where $\text{Δ}Q_{m}$ (in nC/cm^2^) and Δt (in s) are the dose-difference and distance-to-agreement tolerances, respectively.The closest corresponding $Q_{m}^{SONIC}$ point to $\langle Q_{m}^{NICE}\rangle \left(t_{1}\right)$ and its deviation-distance are then obtained by minimizing $\text{Γ}\left(t_{1},\;t_{2}\right)$:

(Equation 21) $$\gamma {\left(t_{1}\right)}_{k}=\min\left(\Gamma {\left(t_{1},\;t_{2}\right)}_{k},\forall \;t_{2}\right)$$

The SONIC gamma deviation $\varepsilon_{\gamma }$ was then defined as the maximal deviation-distance across the entire time range and all compartments:

(Equation 22) $$\varepsilon_{\gamma }=\max(\max\left(\gamma {\left(t\right)}_{k},\forall \;t),\;k\in \left\{A,\;B\right\}\right)$$

Charge density and timing tolerances were expressed as a function of model-specific spiking features (extracted from a single-compartment SONIC simulation at 1.1 times the excitation threshold): the charge difference criterion $\text{Δ}Q_{m}$ and the distance-to-agreement criterion Δt were set to 30% of the spike prominence and spike half-width, respectively.

For both the passive and the axon-specific benchmarks, NICE and SONIC simulations were run using frequency-dependent time steps (*dt*_*NICE*_ = 0.001/*f*_*US*_, *dt*_*SONIC*_ = 1/*f*_*US*_).

### Quantification and statistical analysis

Statistical differences between the firing rate distributions of myelinated and unmyelinated fiber populations in Figure 7C were evaluated using the Kruskal-Wallis test. A resulting p value below 0.05 determined statistical significance.

## Data Availability

• All data reported in this paper will be shared by the lead contact upon request. • All original code has been deposited on GitHub and is publically available at the date of publication. URLs are listed in the key resources table. • Any additional information required to reproduce this work is available from the lead contact upon request.
