# Supplementary material for: MorphoSONIC: A morphologically structured intramembrane cavitation model reveals fiber-specific neuromodulation by ultrasound
Source: iScience. 2021 Sep 6;24(9):103085. doi: 10.1016/j.isci.2021.103085 (PMC8456061; doi:10.1016/j.isci.2021.103085)
Supplement: Document S1. Figures S1 and S2 [file mmc1.pdf]

iScience, Volume *24*

Supplemental information

MorphoSONIC: A morphologically structured intramembrane cavitation model reveals fiber-specific neuromodulation by ultrasound

Théo Lemaire, Elena Vicari, Esra Neufeld, Niels Kuster, and Silvestro Micera

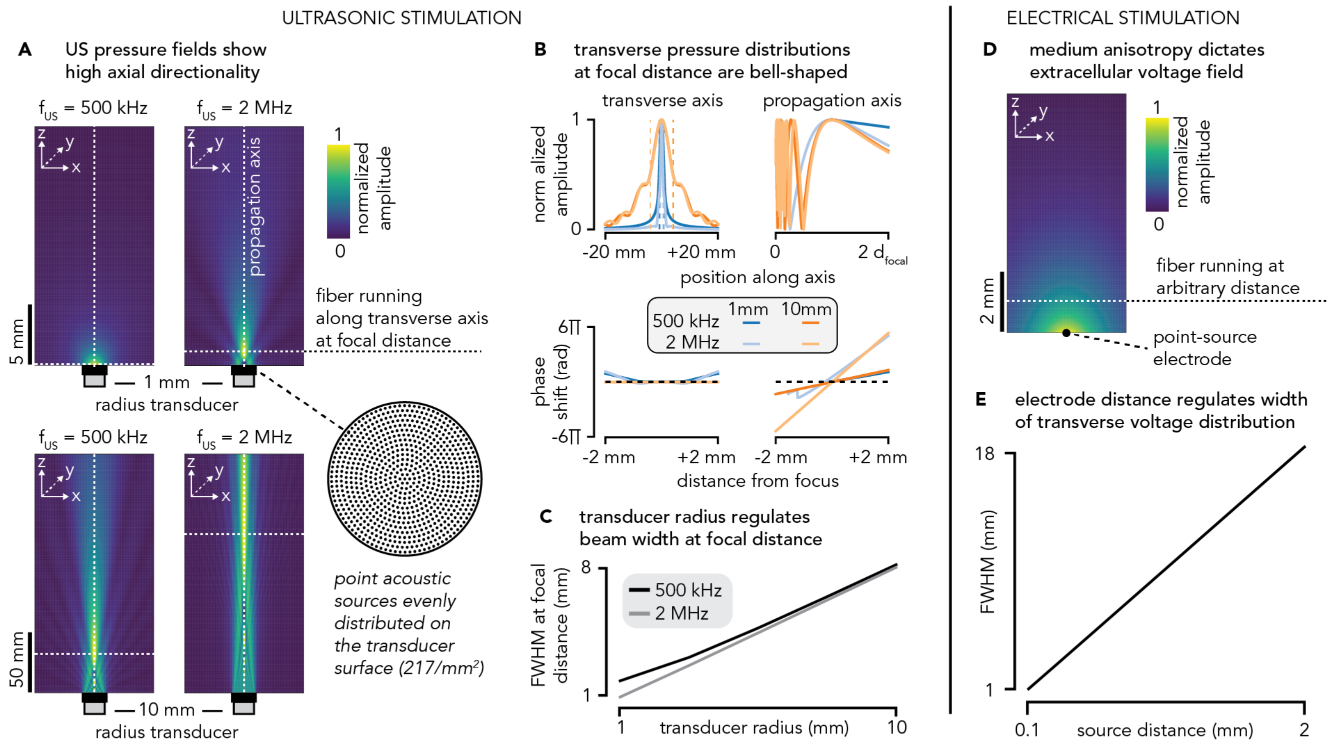

Figure S1. Qualitative nature of exogenous acoustic and electric fields, related to STAR methods. (A) Normalized two-dimensional acoustic pressure phasor amplitude distribution across the propagation plane computed upon sonication by a single-element planar transducer immersed in water-like medium, for various combinations of transducer radius and ultrasound frequency. Reference axes are indicated in white. **(B)** Normalized amplitude and absolute phase distributions of the acoustic pressure field along the transverse (at focal distance) and propagation axes, for the same combinations of transducer radius and ultrasound frequency. Dotted lines indicate FWHMs for each distribution. **(C)** FWHM of the pressure phasor amplitude distribution along the fiber axis as a function of the transducer radius, for two characteristic ultrasound frequencies. **(D)** Normalized two-dimensional voltage distribution across a two-dimensional plane generated by a point-source electrode placed in a nerve-like anisotropic medium. Reference axes are indicated in white. **(E)** FWHM of the extracellular voltage distribution along the fiber axis as a function of the electrode-fiber distance.

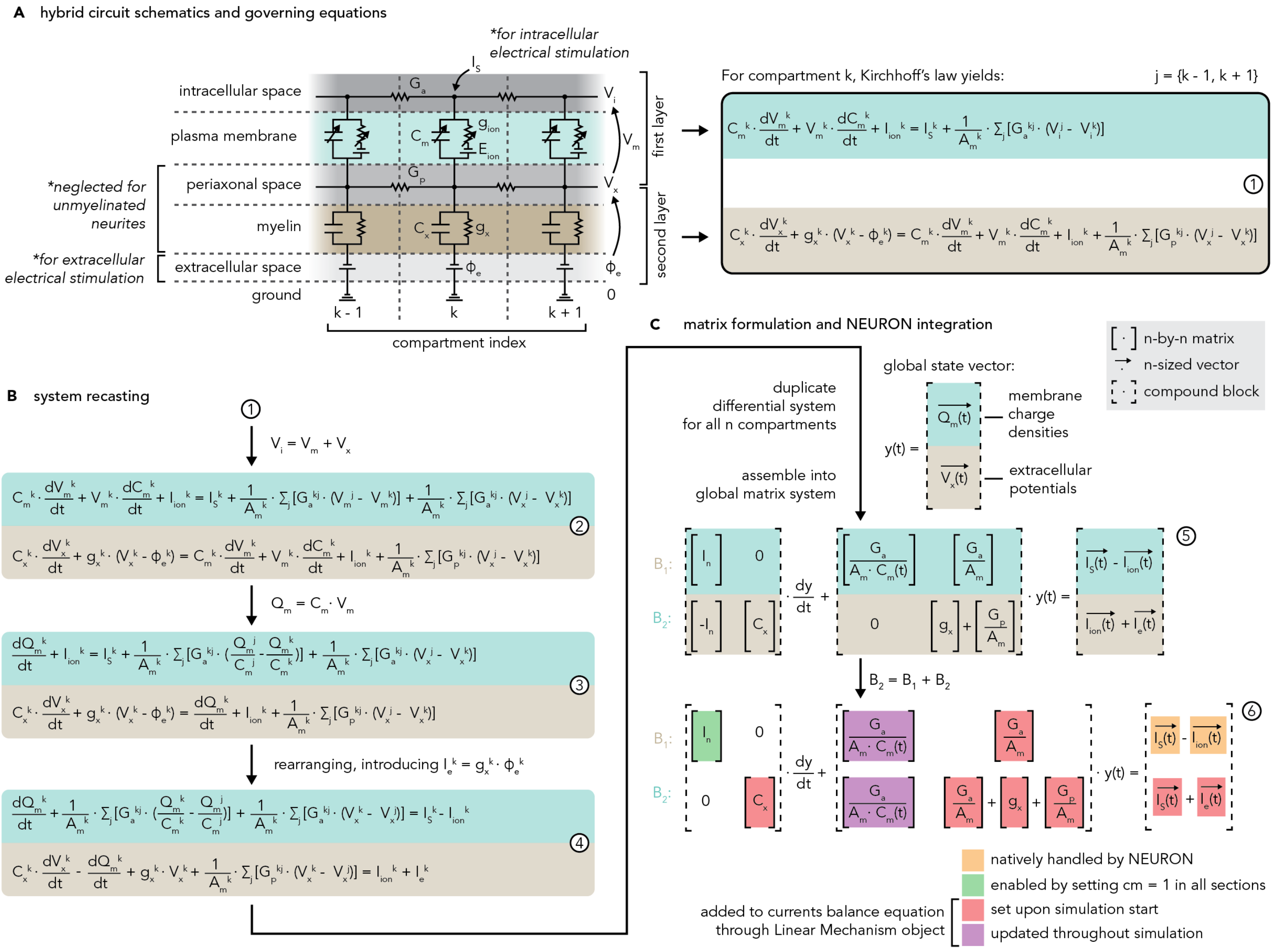

Figure S2. Hybrid multi-compartment, multi-layer electrical circuit for the integration of the charge-casted SONIC model into morphologically-structured models, related to STAR methods. (A) Schematics of the hybrid circuit for a subset of 3 compartments, along with the corresponding governing equations for the central compartment. **(B)** Description of the multiple recasting steps to obtain a SONIC-compatible equation system at the single compartment level. **(C)** Description of the system expansion and assembly into matrix form to cover an arbitrary number of compartments, and of the resolution of this singular matrix system in the NEURON simulation environment.
